# FBXO22 promotes glioblastoma malignant progression by mediating VHL ubiquitination and degradation

**DOI:** 10.1038/s41420-024-01919-2

**Published:** 2024-03-23

**Authors:** Zhigang Shen, Tao Dong, Hongmei Yong, Chuyin Deng, Changxiu Chen, Xintian Chen, Miaolei Chen, Sufang Chu, Junnian Zheng, Zhongwei Li, Jin Bai

**Affiliations:** 1 Cancer Institute, Xuzhou Medical University, Xuzhou, Jiangsu China; 2 Jiangsu Center for the Collaboration and Innovation of Cancer Biotherapy, Cancer Institute, Xuzhou Medical University, Xuzhou, Jiangsu China; 3 Department of Neurosurgery, the Affiliated Hospital of Xuzhou Medical University, Xuzhou, Jiangsu China; 4 Department of Oncology, The Affiliated Huai’an Hospital of Xuzhou Medical University and The Second People’s Hospital of Huai’an, Huaian, Jiangsu China; 5 Department of Pediatrics, the Affiliated Huaihai Hospital of Xuzhou Medical University, Xuzhou, Jiangsu China; 6 Center of Clinical Oncology, the Affiliated Hospital of Xuzhou Medical University, Xuzhou, Jiangsu China; 7 Laboratory of Tumor Epigenetics, School of Basic Medical Sciences, Wannan Medical College, Wuhu, Anhui China; 8 Department of Pathophysiology, School of Basic Medical Sciences, Wannan Medical College, Wuhu, Anhui China

**Keywords:** Ubiquitylation, Oncogenes, CNS cancer, Tumour angiogenesis

## Abstract

Glioblastoma (GBM) is the most common malignant primary brain tumor. Despite comprehensive treatment with traditional surgery, radiotherapy, and chemotherapy, the median survival rate is <14.6% and the 5-year survival rate is only 5%. FBXO22, a substrate receptor of the SCF ubiquitin ligases, has been reported to play a promoting role in melanoma, liver cancer, cervical cancer, and other cancers. However, the function of FBXO22 in GBM has not been reported. In the present study, we demonstrate that FBXO22 is highly expressed in glioma and is positively correlated with worse pathological features and shorter survival of GBM patients. We revealed that FBXO22 promotes GBM cell proliferation, angiogenesis, migration, and tumorigenesis in vitro and in vivo. In terms of mechanism, we reveal that FBXO22 decreases VHL expression by directly mediating VHL ubiquitination degradation, which ultimately increases HIF-1α and VEGFA expression. In addition, our data confirm that there are positive correlations among FBXO22, HIF-1α, and VEGFA expression, and there is a negative correlation between FBXO22 and VHL protein expression in glioma patients. Our study strongly indicates that FBXO22 is a promising diagnostic marker and therapeutic target for glioma patients.

## Introduction

Glioma is the most common primary intracranial malignant tumor in adults [1], and glioblastoma is the most common glioma with the highest degree of malignancy and worst prognosis [2, 3]. The main reasons for the poor prognosis are a large number of abnormal new blood vessels and strong invasiveness [4, 5]. Surgery combined with chemotherapy and radiotherapy is still the standard treatment strategy for GBM patients. With the latest tumor treating fields (TTF), the median survival period is still <2 years [6]. Therefore, it is urgent to find new therapeutic targets.

The ubiquitin-proteasome system (UPS) regulates all cellular processes through precise spatiotemporal control of protein stability, activity, and/or location [7]; it also participates in the degradation of 80% of intracellular proteins, affects genomic stability and signaling pathways, and regulates cell functions, including angiogenesis and invasion [8–10]. E3 ubiquitin ligases play a key role in UPS-mediated protein ubiquitination degradation. Studies have revealed that aberrant expression of a variety of E3 ubiquitin ligases has a great effect on tumorigenesis and cancer metastasis. For instance, our recent reports demonstrate that E3 ligase TRAF6-mediated EZH2 ubiquitination inhibits breast cancer metastasis and prostate cancer progression [11, 12]. E3 ligase TRIM21-mediated HIF-1α and SREBF1 ubiquitination attenuate renal cancer cell glycolysis and lipid metabolism [13].

F-box-only protein 22 (FBXO22) is a ubiquitin ligase that is responsible for recruiting and binding members of the F-box protein family of substrates, participating in the initiation, progression, recurrence, and metastasis of various human malignant tumors [14]. It has been reported that FBXO22 can regulate the malignant progression of several cancers by mediating the degradation of P21 [15], KLF4 [16], KDM4A, and p53 [17]. Our previous study discovered that FBXO22 prevents breast cancer progression by directly catalyzing HDM2 ubiquitination degradation [18]. However, whether FBXO22 affects GBM progression is still unknown.

Hypoxia-inducible factor-1α (HIF-1α) is an alpha subunit of the transcription factor hypoxia-inducible factor (HIF). HIF-1α regulates the transcription of numerous gene types that promote responses to hypoxic conditions, including genes regulating angiogenesis, erythropoiesis, the cell cycle, metabolism, and apoptosis [19]. Vascular endothelial growth factor A (VEGFA) belongs to the PDGF/VEGF family of growth factors and plays a key role in promoting vascular development [20]. Studies have demonstrated that HIF-1α can directly activate VEGFA transcriptional expression. VHL is a classical HIF-1α E3 ligase that mediates its ubiquitination-mediated degradation to maintain very low HIF-1α levels in cells under normoxia. Exposure of cells to hypoxia or loss of VHL function results in increased levels of HIF-1α protein and expression of HIF-induced gene products, mostly angiogenic factors such as vascular endothelial growth factor (VEGF) [21]. The mechanisms of the regulation of VHL expression in GBM are not yet very clear, especially whether some E3 ligases can mediate VHL ubiquitination degradation.

In this study, we first found that FBXO22 is positively correlated with glioma grade and poor prognosis of glioma patients. And we revealed that ectopic expression of FBXO22 promotes glioma cell proliferation, angiogenesis, and cell migration. Mechanistically, we revealed that FBXO22 stimulates the activation of the HIF-1α-VEGFA pathway by directly binding and mediating VHL ubiquitination degradation and promoting the malignant progression of glioblastoma. Finally, we confirmed that there are positive correlations among FBXO22, HIF-1α, and VEGFA expression in glioma patients. Our study suggests that FBXO22 may be a promising therapeutic target for glioma patients.

## Results

### FBXO22 is positively correlated with glioma malignancy and poor prognosis

**Fig. 1:**
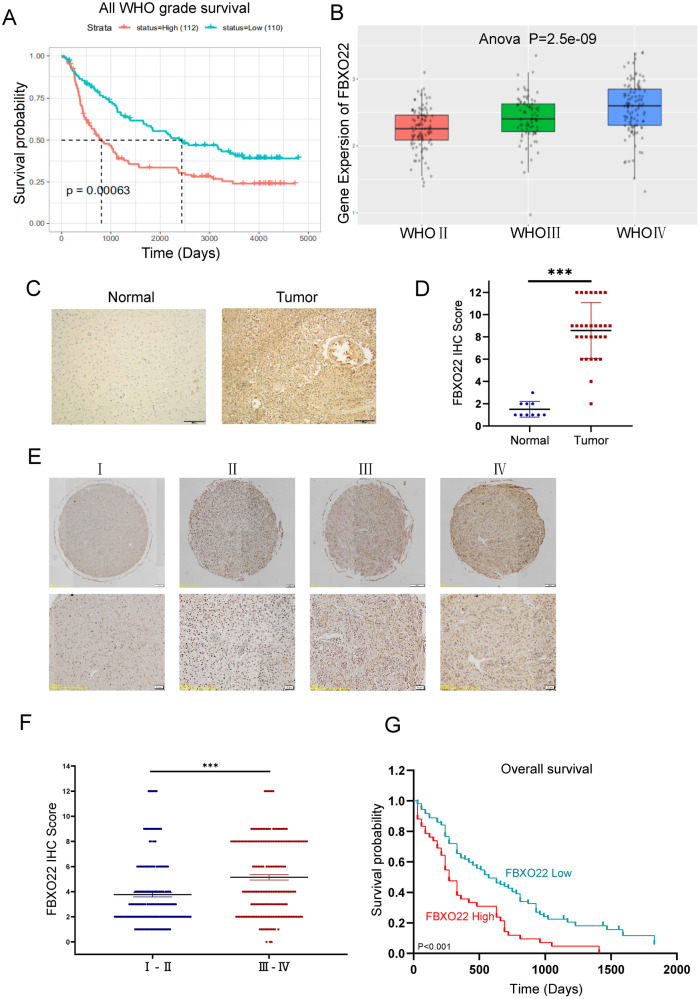
FBXO22 is positively correlated with glioma malignancy and poor prognosis. **A** The survival time of all grades of glioma with different FBXO22 high and low expression levels was found from the TCGA database. **B** FBXO22mRNA expression in different grades of human gliomas as learned from the CGGA database. **C** Representative immunohistochemical images of FBXO22 protein expression in normal and cancerous tissues of glioma patients. **D** Representative immunohistochemical images of FBXO22 protein expression in normal and cancerous tissues of glioma patients. **E** Typical immunohistochemical image of FBXO22 protein expression in patients with different grades of glioma in tissue microarray. **F** FBXO22 protein score statistics of patients with high and low-grade gliomas (*n* = 428, *p* < 0.001) in tissue microarray. **G** Overall survival of glioma patients (*n* = 428, *p* < 0.001) was correlated with FBXO22 protein level by Kaplan–Meier analysis.

To explore the function of FBXO22 in glioma progression, we first analyzed FBXO22 expression in the TCGA database and CGGA database. The data showed that high FBXO22 expression was positively correlated with poor prognosis in primary glioma (Fig. 1A). FBXO22 expression was positively correlated with increasing glioma tumor grade (Fig. 1B). Then, we tested FBXO22 expression in our collected normal human brain tissues and human glioma tissues by IHC assays. We found that FBXO22 was significantly highly expressed in tumor tissues, in line with the outcomes of the TCGA and CGGA analyses (Fig. 1C, D). These data suggest that FBXO22 is highly expressed in glioma tissues compared with normal brain tissues.

Next, we analyzed FBXO22 expression in glioma patients by glioma tissue microarray (TMA), which contains 428 glioma samples with clinical information and prognosis. The IHC assay results revealed that FBXO22 expression was positively correlated with the grade of glioma but not with sex, age, or histological type (Table 1). We found that FBXO22 expression was much higher in high-grade glioma patients (III and IV grades) than in low-grade glioma patients (I and II grades) (Fig. 1E, F). In addition, our data also illustrated that glioma patients with high FBXO22 expression had much shorter survival times than those with low FBXO22 expression (Fig. 1G). Taken together, our data strongly indicate that FBXO22 is correlated with glioma progression and poor overall patient survival.

**Table 1:** FBXO22 staining and clinicopathological characteristics of 428 glioma patients.

Variables	FBXO22 staining			*χ*^2^ value	*p*-value
	Low (%)	High (%)	Total		
All cases	344 (80.4)	84 (19.6)	428		
*Age*					
≤42 years	181 (82.6)	38 (17.4)	219	1.471	0.225
>42 years	163 (78.0)	46 (22.0)	209		
*Gender*					
Male	128 (49.4)	131 (50.6)	259	0.031	0.86
Female	85 (50.3)	84 (49.7)	169		
*WHO grade*					
Benign (I–II)	202 (87.4)	29 (12.6)	231	15.912	<0.001
Malignant (III–IV)	142 (72.1)	55 (27.9)	197		
*Histological type*					
Glioblastoma	18 (72.5)	7 (27.5)	25	4.908	0.427
Astrocytoma	77 (85.6)	13 (14.4)	90		
Oligodendroglioma	11 (78.6)	4 (21.4)	15		
Ependymoma	4 (100)	0 (0)	4		
Medulloblastoma	10 (71.4)	4 (28.6)	14		
Gliocytoma	221 (79.3)	59 (20.7)	280		

### FBXO22 promotes GBM cell proliferation in vitro

**Fig. 2:**
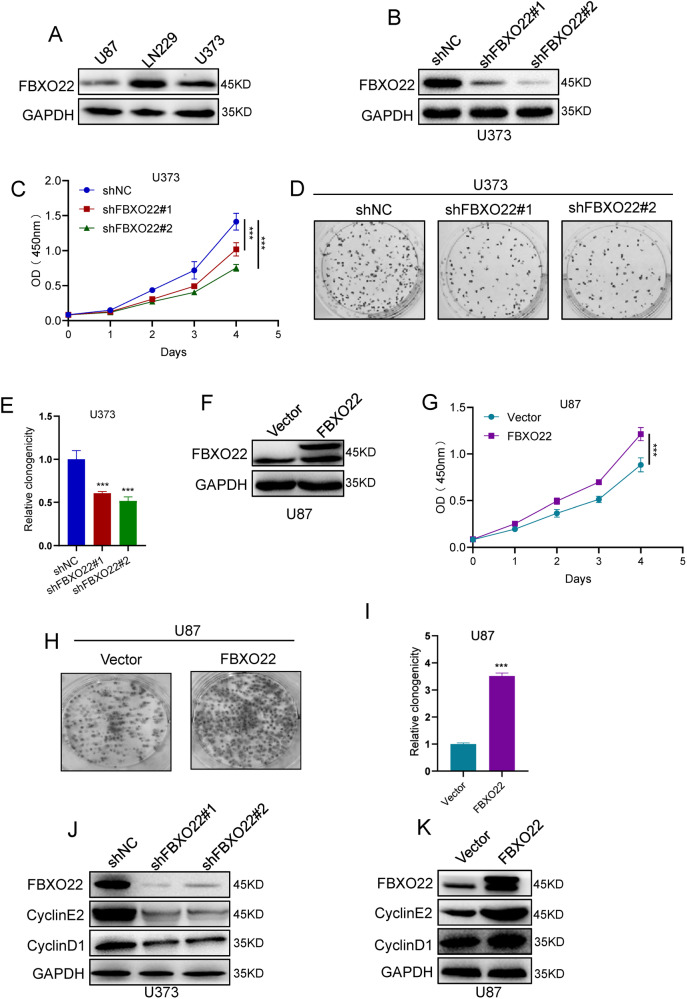
FBXO22 promotes GBM cell proliferation in vitro. **A** Western blot was used to detect the expression of FBXO22 in three cell lines (U87, LN229, and U373). **B**, **F** Knockdown and overexpression efficiency of U373 and U87-FBXO22 were detected by Western blots. **C-E** After knocking down FBXO22 in U373, the cell proliferation ability was detected by CCK8 and colony formation assay. **G–I** CCK8 and colony formation assays were performed after FBXO22 overexpression in U87 cells. **J** and **K** We examined the expression of Cyclin E2 and Cyclin D1 using western blots when FBXO22 was knocked down or overexpressed in U373 and U87 cells. All the results were confirmed by three times repeated experiments. Statistical analysis was performed using unpaired *t*-tests. All statistical tests were two-sided. ***p < 0.001.

Our above results have shown that FBXO22 is highly expressed in glioma patients. We wanted to explore whether FBXO22 affects GBM progression. First, we detected the expression of FBXO22 in three GBM cell lines (U87, LN229, and U373) by Western blot assays. We found that FBXO22 expression was much higher in LN229 and U373 cells than in U87 cells (Fig. 2A). Therefore, we overexpressed FBXO22 in U87 cells and knocked down FBXO22 in LN229 and U373 cells in the following study.

To explore the effect of FBXO22 on proliferation, We knocked down FBXO22 using shRNA in U373 and LN229 cells. Western blot analysis showed that FBXO22 was significantly decreased after silencing FBXO22 (Figs. 2B and S1A). Cell proliferation assays using the CCK-8 kit revealed that the knockdown of FBXO22 inhibited the proliferation of LN229 and U373 cells (Figs. 2C and S1B). Similarly, colony formation assays showed that knockdown of FBXO22 decreased the colony formation ability of LN229 and U373 cells (Figs. 2D, E and S1C, D). On the other hand, we overexpressed FBXO22 in U87 cells and confirmed FBXO22 overexpression by immunoblot assay (Fig. 2F). We found that ectopic expression of FBXO22 enhanced cell proliferation (Fig. 2G) and colony formation (Fig. 2H, I) abilities compared with the control cells.

Moreover, we detected changes in several key cell cycle markers when FBXO22 was knocked down or overexpressed in the abovementioned cells. Our results showed that cyclin-D1 and cyclin-E2 expression was decreased after silencing FBXO22 (Figs. 2J and S3A), whereas cyclin-D1 and cyclin-E2 expression was increased after overexpressing FBXO22 (Fig. 2K). Our data suggest that FBXO22 can promote GBM cell proliferation by increasing cyclin-D1 and cyclin-E2 expression.

### FBXO22 promotes GBM cell motility and angiogenesis in vitro

**Fig. 3:**
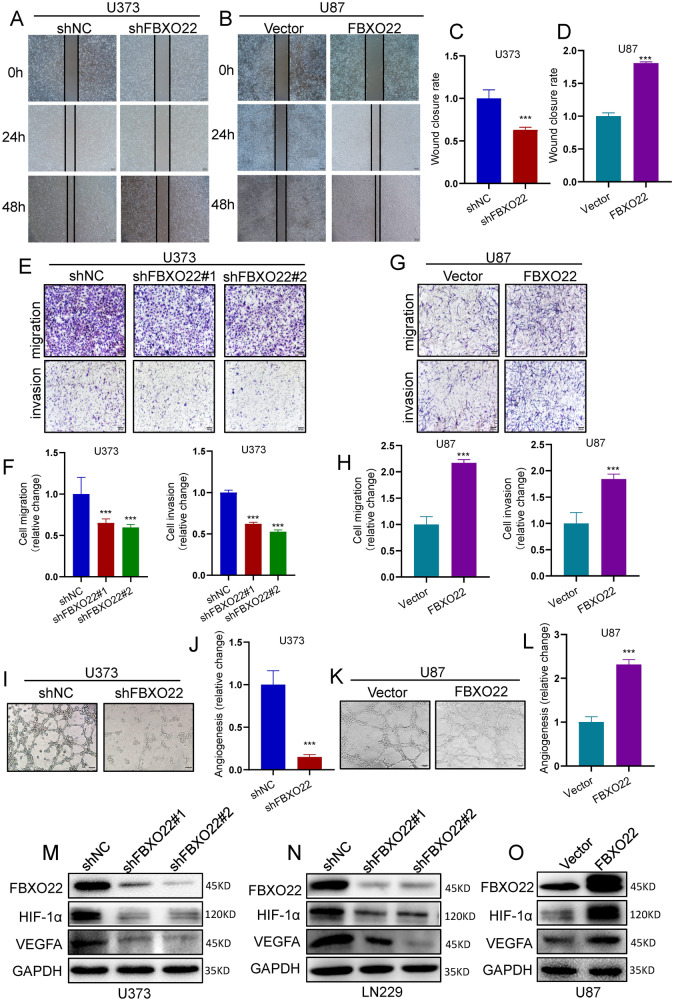
FBXO22 promotes GBM cell motility and angiogenesis ability in vitro. **A–D** Cell motility was assessed by wound-healing assays on FBXO22 knockdown cells and FBXO22 overexpression cells. **E–H** Migration and invasion assays were performed to detect cell motility after the knockdown of FBXO22 in U373 cells and overexpression of FBXO22 in U87 cells, respectively. **I–L** Angiogenesis assay was performed in U373-shNC/shFBXO22 and U87-Vector/FBXO22 to detect the metastatic ability of cells. **M**–**O** After the knockdown of FBXO22 in U373 and LN229 and overexpression of FBXO22 in U87, the expression levels of HIF-1α and VEGFA were detected by western blots. All the results were confirmed by three times repeated experiments. Statistical analysis was performed using unpaired *t*-tests. All statistical tests were two-sided. ***p < 0.001.

Subsequently, we evaluated the role of FBXO22 function in regulating GBM cell migration and invasion. First, our wound-healing assays found that silencing FBXO22 inhibited the motility of U373 cells, while overexpression of FBXO22 enhanced the motility of U87 cells (Fig. 3A–D). Moreover, our transwell assays also confirmed that the knockdown of FBXO22 attenuated LN229 and U373 cell migration and invasion abilities (Figs. 3E, F and S2A-C). Overexpression of FBXO22 facilitated U87 cell motility (Fig. 3G, H).

Many reports have shown that angiogenesis has a great effect on GBM tumorigenesis; for example, Profilin-1 phosphorylation has been reported to guide vasocrine expression through HIF-1α accumulation to promote GBM progression [22]. Therefore, we wondered whether FBXO22 can enhance GBM angiogenesis. Interestingly, our angiogenesis assays indicated that the tube-forming ability of Huvec cells was reduced when treated with the LN229 and U373 cells culture medium after knocking down FBXO22. Conversely, the tube-forming ability was increased when Huvec cells were cultured with U87 cells medium after overexpressing FBXO22 (Figs. 3I–L and S2D, E). These results suggest that FBXO22 promotes the angiogenic capacity of GBM.

We then investigated the mechanisms by which FBXO22 promoted GBM angiogenesis. We examined several classical drivers in GBM angiogenesis formation. We revealed that HIF-1α and VEGFA were dramatically downregulated after silencing FBXO22 in LN229 and U373 cells (Fig. 3M, N). At the same time, we also found that HIF-1α and VEGFA expression were elevated when FBXO22 was ectopically expressed in U87 cells (Fig. 3O). Taken together, our data indicate that FBXO22 may promote GBM cell motility and angiogenesis by activating the HIF-1α-VEGFA axis.

### FBXO22 promotes HIF-1α and VEGFA expression by directly mediating VHL ubiquitin degradation

**Fig. 4:**
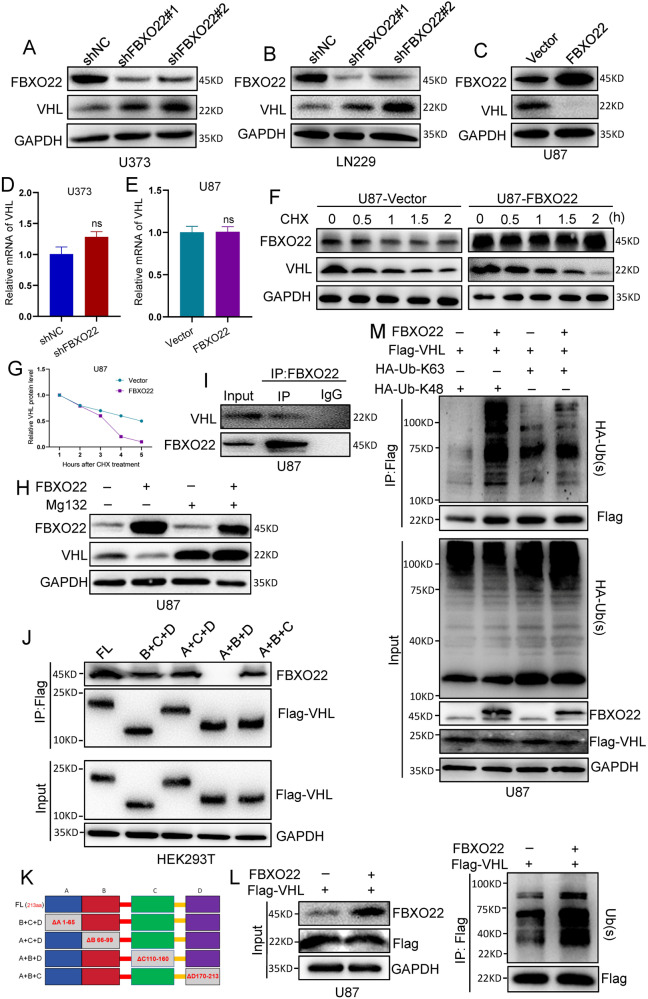
FBXO22 promotes HIF-1α and VEGFA expression by directly mediating VHL ubiquitin degradation. **A–C** The protein expression of VHL in U373-shNC/shFBXO22, LN229-shNC/shFBXO22, and U87-Vector/FBXO22 cells was detected. **D**, **E** VHL mRNA expression in FBXO22 knockdown cells and FBXO22 overexpression cells was assessed by qRT-PCR assay. **F**, **G** After treatment with CHX, VHL protein expression was detected in FBXO22 overexpression, and the relative intensity of VHL protein was quantified using the software ImageJ. **H** After treatment with MG132 for 4 h, VHL protein expression in U87-Vector/FBXO22 cells was detected by western blots. **I** Western blots analysis of FBXO22 binding to VHL after IP HA in U87 after overexpression of HA-FBXO22 and Flag-VHL. **J** IP Flag was performed in 293T cells after transfection of the missing mutant with the corresponding domain, and Western blots analysis was used to detect the domain of VHL and FBXO22 interaction. **K** According to the website of Uniport (www.uniport.org), four missing bodies, as shown in the figure are constructed. **L** Western blots detection of VHL-associated ubiquitination after IP Flag in HA-FBXO22 and Flag-VHL overexpression cells. **M** HA-Ub-K48 and HA-Ub-K63 plasmids alone or cotransfected with HA-FBXO22 plasmid and Flag-VHL plasmid into U87 cells for 48 h, and the cells were then harvested and subjected to Flag IP. All the results were confirmed by three times repeated experiments. Statistical analysis was performed using unpaired *t*-tests. All statistical tests were two-sided. ns, no significance.

Moreover, we wanted to determine the detailed mechanism by which FBXO22 elevates HIF-1α expression. Many reports have demonstrated that VHL, as an E3 ligase, can decrease HIF-1α expression by directly mediating its ubiquitination. Interestingly, our Western blot analysis showed that silencing FBXO22 strongly increased VHL expression at the protein level (Fig. 4A, B), while overexpression of FBXO22 dramatically decreased VHL protein expression (Fig. 4C). Furthermore, our data showed that the mRNA expression of VHL was not significantly changed regardless of whether FBXO22 was knocked down or overexpressed (Figs. 4D, E and S2F, G). The above data suggest that FBXO22 may regulate VHL expression at the posttranslational level.

Since FBXO22 is an E3 ligase, we speculate that FBXO22 may catalyze VHL ubiquitination degradation to attenuate its protein expression, which prevents VHL-mediated HIF-1α degradation and ultimately promotes HIF-1α and VEGFA expression. To validate our hypothesis, we first detected the VHL protein half-life in U87-Vector and U87-FBXO22 cells after treatment with cycloheximide (CHX) for different durations. The results showed that overexpression of FBXO22 reduced the protein half-life of VHL (Fig. 4F, G). Then, our Western blot assays showed that FBXO22 overexpression significantly increased VHL protein expression in U87 and U373 cells after treatment with the proteasome inhibitor MG132 (Figs. 4H and S3B). These observations strongly suggest that FBXO22 can regulate VHL protein stability through the ubiquitin-proteasome pathway.

We subsequently performed co-IP assays in HEK293T and U87 cells. Our results revealed both exogenous and endogenous FBXO22-VHL interactions in U87 cells and HEK293T cells, respectively (Figs. 4I and S4A–C). Next, we conducted a website analysis to identify the four domains of VHL, which were named A–D (Fig. 4K). To investigate the domains necessary for the interaction and degradation of FBXO22 and VHL, we generated deletion plasmids lacking the corresponding domains. We performed IP assays after these mentioned plasmids were transfected into HEK293T cells respectively. The results indicated that deletion mutants lacking the C domain exhibited an impaired ability to interact with FBXO22, suggesting that the C domain is primarily necessary for VHL–FBXO22 interaction (Fig. 4J). Moreover, we investigated the changes in VHL ubiquitination after ectopic expression of FBXO22 in HEK293T cells and U87 cells. The data indicated that the amount of VHL ubiquitination was strongly elevated in these cells after overexpressing FBXO22 (Figs. 4L and S4D). Besides, we also measured the ubiquitin level of HIF-1α in overexpressing FBXO22 and Vector U87 cells. The results revealed that the amount of HIF-1α ubiquitination was significantly decreased after ectopic expression of FBXO22 (Fig. S4E). It suggests that FBXO22-mediated VHL ubiquitination probably can repress VHL-mediated HIF-1α ubiquitination through decreasing VHL stability, which results in FBXO22 increased HIF-1α expression in GBM cells.

Additionally, in order to determine the detailed mechanism of FBXO22-mediated VHL ubiquitination degradation, we used different types of HA-ubiquitin mutants (K6, K11, K27, K29, K33, K48, and K63) to conduct ubiquitination experiments. Firstly, our IP-Flag-VHL assays showed that the K48-linked ubiquitin chain had higher levels of ubiquitin in HEK293T cells after co-expressing HA-ubiquitin and Flag-VHL plasmids (Fig. S5A). Then, we transfected HA-Ub-K48 plasmid or HA-Ub-K63 plasmid with Flag-VHL plasmid into U87 cells to detect the detailed ubiquitin type of FBXO22-mediated VHL ubiquitination by IP assays in GBM cells (Fig. 4M). Our results revealed that FBXO22 mainly catalyzes VHL K48-linked ubiquitination. Above all, our results demonstrate that FBXO22 mediates VHL K48-linked ubiquitination and decreases its protein stability in GBM cells.

### Decreasing VHL expression is necessary for FBXO22 to promote GBM cell proliferation, migration, and angiogenesis

**Fig. 5:**
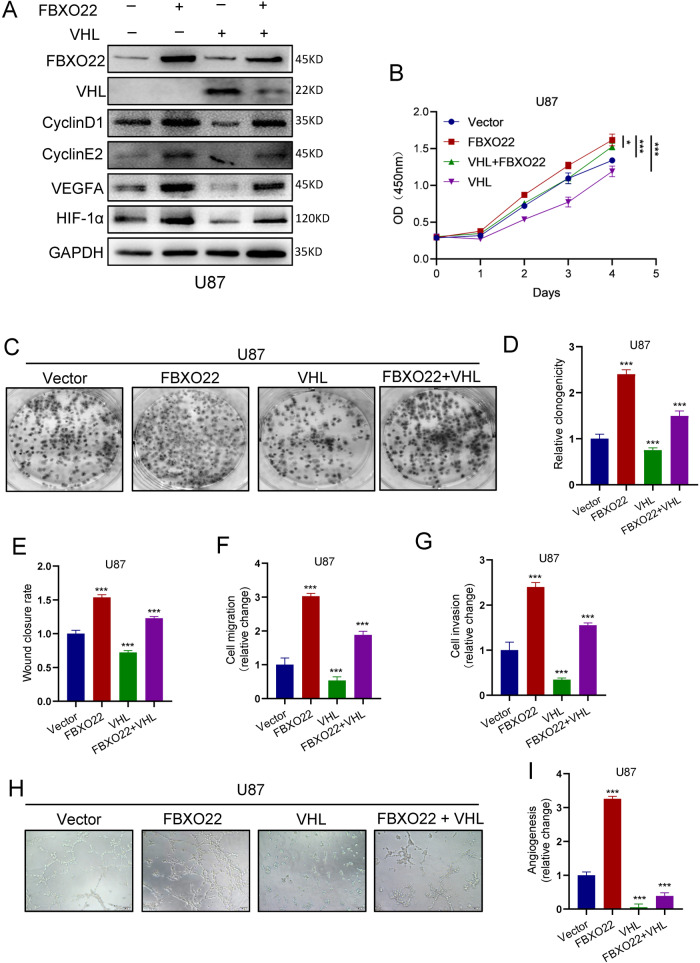
Decreasing VHL expression is necessary for FBXO22 to promote GBM cell proliferation, migration, and angiogenesis. **A** Western blot was used to detect the protein expression of HIF-1α, VEGFA, Cyclin E2, and Cyclin D1 in U87-Vector/FBXO22/VHL/FBXO22 + VHL cells. **B–D** CCK8 and colony formation assays were used to analyze the cell proliferation capacity of U87-Vector/FBXO22/VHL/FBXO22 + VHL cells. **E–G** The migration and invasion abilities of these cells were examined by wound healing and Transwell assay. **H**, **I** The metastatic capacity of U87-Vector/FBXO22/VHL/FBXO22 + VHL cells was tested by angiogenesis assay. All the results were confirmed by three times repeated experiments. Statistical analysis was performed using unpaired *t*-tests. All statistical tests were two-sided. *p < 0.05, **p < 0.01, ***p < 0.001.

To elucidate the role of FBXO22-mediated VHL degradation in HIF-1α and VEGFA promotion of GBM progression, we performed rescue experiments by ectopically expressing VHL in U87-FBXO22 cells. Our results showed that the expression levels of HIF-1α, VEGFA, Cyclin E2, and Cyclin D1 were decreased when VHL was overexpressed in U87-FBXO22 cells (Fig. 5A). CCK8 assays and colony formation assay data showed that cell proliferation ability was weakened when VHL was overexpressed in U87-FBXO22 cells (Fig. 5B–D).

Moreover, we also found that overexpression of VHL in U87-FBXO22 cells inhibited their migration and invasion abilities by Transwell experiments (Figs. 5E–G and S6A, B). Next, we overexpressed HIF-1α in U373-shFBXO22 cells and performed rescue experiments. The experimental results demonstrated that the overexpression of HIF-1α in U373-shFBXO22 cells significantly enhanced cell proliferation, migration, and invasion (Fig. S7A–G). These results suggest that downregulation of VHL is required for FBXO22 to promote GBM cell proliferation, migration, and invasion.

In addition, we also performed angiogenesis assays to illustrate whether FBXO22 facilitates GBM cell angiogenesis via the VHL–HIF-1α–VEGFA signaling axis. Our rescue results showed that the medium used to culture FBXO22- and VHL-co-expressing U87 cells resulted in a significant reduction in angiogenesis compared with only FBXO22-overexpressing U87 cells (Fig. 5H, I). These results suggest that FBXO22-mediated VHL degradation is required for FBXO22 to enhance GBM cell angiogenesis.

### The FBXO22–VHL–HIF-1α–VEGFA axis promotes GBM tumorigenesis in vivo

**Fig. 6:**
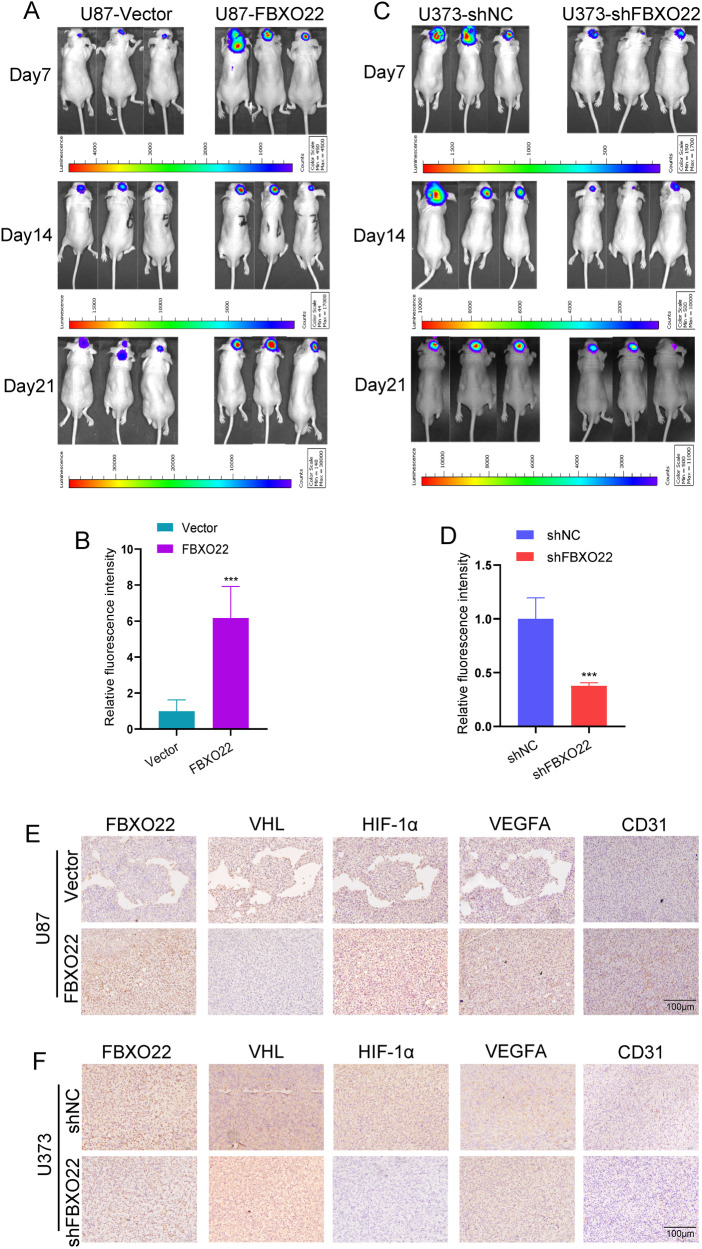
FBXO22–VHL–HIF-1α–VEGFA axis promotes GBM tumorigenesis in vivo*.* **A–D** U87-Vecotr/FBXO22 and U373-shNC/shFBXO22 cells were orthotopic implanted into the right striatum of each mouse, respectively, *n* = 5 in each group. The effect of FBXO22 on the orthotopic glioma cell implantation model was evaluated by evaluating the fluorescence intensity of the tumors. **E**, **F** The expressions of FBXO22, VHL, HIF-1α, VEGFA, and CD31 in orthotopic implantation tumors were detected by IHC. Statistical analysis was performed using unpaired *t*-tests. All statistical tests were two-sided. ***p < 0.001.

Next, we investigated the effect of FBXO22 on GBM tumor growth in vivo. On the one hand, the same number of U87-Vector and U87-FBXO22 cells were injected orthotopically into the brains of nude mice, and the immunofluorescence intensity was detected and recorded every 7 days. The mice were sacrificed after 3 weeks, and the brains of the mice were removed for IHC. As shown in the data, the fluorescence intensity of U87-FBXO22 was significantly stronger than that of the U87-Vector group (Fig. 6A, B). On the other hand, the same number of U373-shNC and U373-shFBXO22 cells were injected into the brains of nude mice, and the same methods were used to observe tumor growth in vivo. Our data showed that the fluorescence intensity of U373-shFBXO22 was much weaker than that of the U373-shNC group (Fig. 6C, D). These data indicate that FBXO22 can promote GBM tumorigenesis in vivo.

Correspondingly, IHC analysis of the removed mouse brain tumor sections confirmed the high expression of FBXO22, HIF-1α and VEGFA and low expression of VHL in the U87-FBXO22 group compared with the U87-Vector group (Fig. 6E). The IHC staining results of the U373-shFBXO22 group were the opposite, i.e., FBXO22, HIF-1α and VEGFA levels were decreased and VHL levels were increased in the U373-shFBXO22 group (Fig. 6F). At the same time, CD31, the classical angiogenesis marker, was also detected by IHC in these mouse-formed brain tumors. The staining results showed that the expression levels of CD31 were increased in the FBXO22-overexpressing tumor group (Fig. 6E), while its expression in the shFBXO22 tumor group was decreased compared with that in the shNC tumor group (Fig. 6F). These in vivo data suggested that FBXO22-mediated VHL degradation can promote GBM tumor growth. Taken together, our data suggest that the FBXO22–VHL–HIF-1α–VEGFA signaling axis may play a key role in GBM tumorigenesis in vivo.

### The FBXO22–VHL–HIF-1α–VEGFA cascade is correlated with glioma patient clinicopathological characteristics

**Fig. 7:**
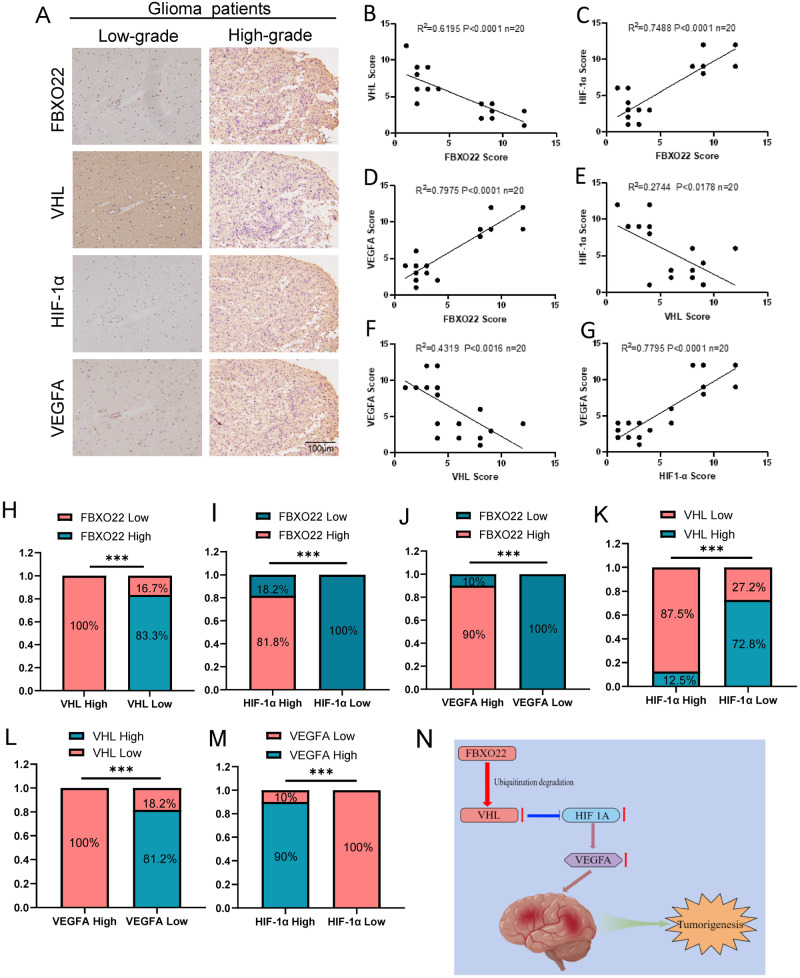
The FBXO22–VHL–HIF-1α–VEGFA cascade is correlated with glioma patient clinicopathological characteristics. **A** Representative images of FBXO22, VHL, HIF-1α, and VEGFA expression in low-grade and high-grade tumors. **B–G** The expression of FBXO22, VHL, HIF-1α, and VEGFA in glioma specimens was detected by IHC, and the scores of FBXO22, VHL, HIF-1α, and VEGFA IHC staining were analyzed by semi-quantitative scoring method (on a scale from 0 to 12). **H–M** Fisher’s exact test was used for the expression of FBXO22 and VHL, FBXO22 and HIF-1α, FBXO22 and VEGFA, VHL and HIF-1α, VHL and VEGFA and HIF-1α and VEGFA, respectively. **N** A cartoon summarizes our findings: FBXO22 degrades VHL by ubiquitination, which results in elevated expression of HIF-1α and VEGFA. The FBXO22-VHL-HIF-1α-VEGFA cascade leads to GBM tumorigenesis. ***p < 0.001.

Finally, we wanted to explore the clinical significance of FBXO22 and VHL functions in glioma. The expression of FBXO22, VHL, HIF-1α, and VEGFA was detected by IHC staining using anti-FBXO22, VHL, HIF-1α, and VEGFA antibodies in the tissue sections of glioma patients. Our results showed that the expression levels of FBXO22, HIF-1α, and VEGFA in high-grade gliomas were much higher than those in low-grade gliomas, while the expression level of VHL in high-grade gliomas was much lower than that in low-grade gliomas (Fig. 7A).

Moreover, we found that FBXO22 was negatively correlated with VHL expression but positively correlated with HIF-1α and VEGFA expression (Fig. 7B–D). In addition, we discovered that VHL was negatively correlated with the expression of HIF-1α and VEGFA (Fig. 7E, F). We also found that there was a positive correlation between HIF-1α and VEGFA (Fig. 7G). The above data suggested a clear link between the FBXO22–VHL–HIF-1α–VEGFA axis and the clinicopathological features of human gliomas.

## Discussion

Accumulating evidence indicates that FBXO22 plays an essential role in the progression of human cancer [14]. However, the influence of FBXO22 on the malignant progression of GBM and potential molecular research is still lacking. In the present study, we reported for the first time that FBXO22-mediated increases in HIF-1α and VEGFA expression can promote GBM cell viability and angiogenesis by binding and degrading VHL, revealing a new mechanism by which FBXO22 promotes GBM malignant progression.

Glioblastoma (GBM) is one of the most highly vascularized human cancers [23]. Since tumor necrosis and microvascular proliferation are pathological features of GBM, tumor blood vessels in GBM are considered major targets for drug development [24]. For instance, bevacizumab has antitumor effects by targeting vascular endothelial growth factor (VEGF) and inhibiting angiogenesis [25]. VEGFA is primarily known for its role in promoting angiogenesis [20]. In this study, we found that the expression of HIF-1α and VEGFA increased, and the expression of VHL decreased in the tissues of patients with high expression of FBXO22. We also demonstrated that FBXO22 was positively correlated with CD31 (angiogenesis marker), HIF-1α and VEGFA expression in xenograft GBM tumors, which proved that FBXO22 accelerated GBM angiogenesis and tumorigenesis. We illustrated that the C domains of VHL mainly interact with FBXO22, and FBXO22 facilitated HIF-1α and VEGFA expression by directly mediating VHL K48-linked ubiquitination degradation. The decrease in VHL attenuated VHL-mediated HIF-1α degradation, which resulted in high HIF-1α and VEGFA expression in GBM cells. Our findings suggest that inhibiting FBXO22 expression might become an antiangiogenic therapeutic strategy for GBM patients.

FBXO22, an F-box receptor subunit of the SCF E3 ligase, has recently been considered to play a key role in many aspects related to cancer development and therapeutic response [26]. Some key substrates of FBXO22 have been found in a variety of cancers. For example, FBXO22 selectively ubiquitinates PTEN in the nucleus to accelerate its degradation by the proteasome and promote the occurrence of colon cancer [27]. FBXO22 promotes the pathogenesis and progression of hepatocellular carcinoma by mediating ubiquitination and degradation of p21 [15], promotes the progression of liver cancer by mediating polyubiquitination and degradation of KLF4 [16], promotes the growth of lung cancer cells by mediating polyubiquitination and inactivation of LKB1 [28], and promotes the progression of cervical cancer by targeting p57Kip2 [29]. These reports mainly showed that FBXO22 promotes malignant cancer progression by mediating its substrate ubiquitination degradation. However, other studies have also demonstrated that FBXO22 can inhibit the progression of many cancers by catalyzing its substrate degradation. For instance, our recent report revealed that FBXO22 targets HDM2 and mediates its ubiquitination degradation, which prevents breast cancer cell invasion and metastasis [18]. In addition, reports have also discovered that FBXO22 inhibits human renal cancer metastasis and makes non-small cell lung cancer (NSCLC) cells sensitive to ionizing radiation (IR) and cisplatin [30]. In this study, we found that high expression of FBXO22 has stronger angiogenic and invasive ability and is associated with a worse prognosis, i.e., FBXO22 is able to promote glioblastoma malignant progression. The above studies showed that FBXO22 can not only promote cancer progression but also inhibit cancer progression. We speculate that the different functions of FBXO22 might depend on the changes in microenvironments in different cancers.

## Conclusions

Our study demonstrates that FBXO22 promotes GBM cell angiogenesis and subsequent GBM malignant progression. We illustrate that these functions depend on FBXO22 activating the HIF-1α–VEGFA pathway by directly catalyzing VHL ubiquitination degradation. Our study suggests that inhibiting FBXO22 expression is a potential intervention strategy for glioblastoma patients.

## Methods

### Cell culture and treatment

Human umbilical vein endothelial cells (Huvec) and human embryonic kidney 293T (HEK293T) cells were obtained from the Cell Bank of China Science Academy (Shanghai, China). The U87 cells, U373 cells and LN229 cells were purchased from American Type Culture Collection.U87, U373, LN229, HUVEC, and HEK293T cell lines were maintained in DMEM (KGM12800NH-500, Keygen Biotech, China) supplemented with 10% fetal bovine serum (BC-SE-FBS01, Biochannel, China) at 37 °C, in 5% CO_2_ humid atmosphere.

All plasmids were purchased from GenePharma Technology (Shanghai, China). Lipofectamine 2000 (Life Technologies, Carlsbad, CA, USA) was used for plasmid transfection according to the instructions of the product side.

### Patients and sample collection

The human tissue study was approved by the Ethics Committee of the Affiliated Hospital of Xuzhou Medical University, China, and was conducted in accordance with the Declaration of Helsinki. Tissue microarray (TMA) slides of 428 glioma tissues, 4 normal brain tissues, and 20 glioma tissues were registered in the Affiliated Hospital of Xuzhou Medical University from 2016 to 2021. The clinicopathological data of the patients were obtained from the medical records of the Affiliated Hospital of Xuzhou Medical University.

### Western blot analysis and antibodies

Western blot assay was performed as detailed in the previous article [31]. Antibody details were as follows: VHL (#68547, Cell Signaling Technology), FBXO22 (13606-1-AP, Proteintech), HIF-1α (11587-1-AP, Proteintech), GAPDH (60004-1-AP, Proteintech), VEGF (19003-1-AP, Proteintech), Cyclin D1 (2978T, Cell Signaling Technology), Cyclin E2 (4132T, Cell Signaling Technology), Flag tag (66008-4-Ig, Proteintech), HA tag (51064-2-AP, Proteintech), Ubiquitin (#3936, Cell Signaling Technology. All Western blot experiments were repeated a minimum of three times.

### Immunohistochemistry (IHC) and antibodies

The experimental modalities for IHC are detailed in our previous article [32]. For the primary antibody, anti-FBXO22 antibody and anti-VEGF antibody were used at a 1:200 dilution. Anti-VHL antibody and anti-HIF antibody were diluted at 1:100. Anti-CD31 antibody (11265-1-AP, Proteintech) was diluted at 1:5000.

### RNA extract, reverse transcription, and qRT-PCR

RNA was extracted from different cell lines using TRIzol (Invitrogen) and reverse transcribed into cDNA using HiScript QRT SuperMix for qPCR (Vazyme Biotech, Nanjing, China). The cDNA products were analyzed by qRT-PCR in LightCycler96. Detailed procedures are described in our previous article [33]. The PCR primers were as follows:

FBXO22 forward:5′-CGGAGCACCTTCGTGTTGA-3′

Reverse: 5′-CACACACTCCCTCCATAAGCG-3′

VHL forward: 5′-CCCGGATCATCATCTGCAATC-3′

Reverse: 5′-AAGGGTGGCTTCGGAAGTTG-3′

GADPH forward: 5’-GACAAGCTTCCCGTTCTCAG-3′

Reverse: 5’-GAGTCAACGGATTTGGT CGT-3′

### Cell proliferation, migration, invasion, and wound-healing assays

Cell proliferation was detected by CCK8 assay after cell counting according to the instructions of the CCK8 kit (CCK-8; Keygen Biotech, China). Cell migration, invasion assay, and wound-healing assays were performed as previously described [34].

### Angiogenesis assay

After transfection, U87 and U373 were cultured in DMEM supplemented with 10% fetal bovine serum for 24 h, the medium was collected, and the supernatant was collected after centrifugation to avoid any cells or cell debris. A total of 20,000 Huvecs were suspended in 100 μL of complete medium and added to 96-well plates laid through Matrigel (BD Biosciences), and cultured at 37 °C for 4 h.

### Stable cell line generation

Cells were formed by lentivirus (GenePharma) infection to form stable cells, and Puromycin was used for screening 6–9 h after infection. Specific methods as detailed in our previous paper [35].

### GBM intracranial mouse model

All experimental protocols were performed in accordance with the guidelines for animal experimental research of Xuzhou Medical University, which were approved by the Institutional Animal Use Committee. Five-week-old male nude mice were purchased from the GemPharmatech Company to establish intracranial GBM xenografts. A total of 8 × 10^5^ U373 stable cell lines and 5 × 10^5^ U87 stable cells were injected into the right striatum of mice, respectively. Cells were transfected with luciferase. Bioluminescence imaging was performed on day 7, day 14, and day 21 after implantation to monitor the growth of intracranial tumors. Detailed experimental procedures for the establishment of the in vivo model are described in our previous article [36]. Mice were sacrificed when they developed cachexia, hemiplegia, delirium, and other neurological symptoms.

### Available clinical data analysis

The clinical data of FBXO22 were searched from the Cancer Genome Atlas (TCGA, https://portal.gdc.cancer.gov) and Chinese glioma Genome Atlas (CGGA, http://www.cgga.org.cn/).

### Statistical analysis

SPSS 20.0 software (SPSS Inc., Chicago, IL, USA) and GraphPad Prism 8 made use of statistical analysis. All data are presented as the means ± SEM, and *p* < 0.05 were considered to indicate statistical significance. The chi-square test was used to assess the related factor analysis of FBXO22 and glioma patients, and Kaplan–Meier and log-rank tests were used to evaluate the correlation between FBXO22 expression and survival of glioma patients. Student’s *t*-test was utilized to analyze the differences between different groups.

### Supplementary information

Supplementary Information

Supplementary Figure 1

Supplementary Figure 2

Supplementary Figure 3

Supplementary Figure 4

Supplementary Figure 5

Supplementary Figure 6

Supplementary Figure 7

Original Data File

## Data Availability

The data supporting the findings of this study are available from the corresponding author upon reasonable request.
