# Supplementary material for: FBXO22 promotes glioblastoma malignant progression by mediating VHL ubiquitination and degradation
Source: Cell Death Discov. 2024 Mar 23;10:151. doi: 10.1038/s41420-024-01919-2 (PMC10959977; doi:10.1038/s41420-024-01919-2)
Supplement: Supplementary file 1 — Supplementary Information [file 41420_2024_1919_MOESM1_ESM.docx]

FBXO22 promotes glioblastoma malignant progression by mediating VHL ubiquitination degradation

Zhigang Shen^#^^1,4^, Tao Dong^#1,4^, Hongmei Yong^#5^, Chuyin Deng^1^, Changxiu Chen^6^, Xintian Chen^1^, Miaolei Chen^1^, Sufang Chu^1^, Junnian Zheng*^1,2,3^, Zhongwei Li*^1,2,3^ and Jin Bai*^1,2,3^

^1^Cancer Institute, Xuzhou Medical University, Xuzhou, Jiangsu, China.

^2^Center of Clinical Oncology, the Affiliated Hospital of Xuzhou Medical University, Xuzhou, Jiangsu, China.

^3^Jiangsu Center for the Collaboration and Innovation of Cancer Biotherapy, Cancer Institute, Xuzhou Medical University, Xuzhou, Jiangsu, China.

^4^Department of Neurosurgery, the Affiliated Hospital of Xuzhou Medical University, Xuzhou, Jiangsu, China.

^5^Department of Oncology, The Affiliated Huai'an Hospital of Xuzhou Medical University and The Second People's Hospital of Huai'an, Huaian, Jiangsu, China

^6^Department of Pediatrics, the Affiliated Huaihai Hospital of Xuzhou Medical University, Xuzhou, Jiangsu, China.

**Authorship notes:** #These authors contributed equally to this work.

**Running title**: FBXO22 promotes GBM malignant progression.

***Correspondence Authors:**

Jin Bai, Cancer Institute, Xuzhou Medical University. 209 Tongshan Road, Xuzhou, 221004, Jiangsu Province, China. E-mail: bj@xzhmu.edu.cn.

Zhongwei Li, Cancer Institute, Xuzhou Medical University. 209 Tongshan Road, Xuzhou, 221004,Jiangsu Province, China. E-mail: lizw074@xzhmu.edu.cn.

Junnian Zheng, Cancer Institute, Xuzhou Medical University. 209 Tongshan Road, Xuzhou, 221004, Jiangsu Province, China. E-mail: jnzheng@xzhmu.edu.cn.

**Conflict of interest:** No potential conflicts of interest were disclosed by the authors.

**Supplementary Methods**

The staining scores of FBXO22, VHL, HIF-1α, VEGFA and CD31 were blindly and independently performed by different pathologists. The signal was quantified based on the degree of positive cell staining and the percentage of staining. Staining intensity was scored from 0 to 3 (0= negative, 1= weak, 2= moderate, 3= strong), and the percentage of positively stained cells was also divided into four categories: 1 (0% to 25%), 2 (26% to 50%), 3 (51% to 75%), and 4 (76% to 100%). The IHC score levels of FBXO22, VHL, HIF-1α, VEGFA and CD31 staining were detected by IRS. The IRS score was calculated by comparing the staining intensity score with the percentage of positive cells. According to IRS, they were divided into low (IRS:0-6) and high (IRS:8-12).

**Supplementary Figure Legends**

**Figure S1** A The knockdown efficiency of LN229FBXO22 was detected by Western blots. B-D After knockdown of FBXO22 in LN229, cell proliferation ability was measured by CCK8 and colony formation assays.All the results were confirmed by three times repeated experiments. Statistical analysis was performed using unpaired t-tests. All statistical tests were two-sided. **p* < 0.05, ***p* < 0.01, ****p*< 0.001.

**Figure S2** A-C Migration and invasion assays were performed to detect cell motility After knockdown of FBXO22 in LN229 cells. D, E Angiogenesis assay was performed in LN229-shNC/shFBXO22 to detect the metastatic ability of cells. F, G FBXO22 mRNA expression in FBXO22 knockdown cells and FBXO22 overexpression cells was assessed by qRT-PCR assay. All the results were confirmed by three times repeated experiments. Statistical analysis was performed using unpaired t-tests. All statistical tests were two-sided. **p* < 0.05, ***p* < 0.01, ****p*< 0.001.

**Figure S3** A When FBXO22 was knocked down in LN229, the expression of CyclinE2 and CyclinD1 was measured using western blots. B After treatment with MG132 for 4h, VHL protein expression in U373-Vector/FBXO22cells was detected by western blots.

**Figure S4** A-C Western blots analysis of FBXO22 binding to VHL after IP HA or IP Flag in U87 and HEK293T after overexpression of HA-FBXO22 and Flag-VHL. D Western blots detection of VHL-associated ubiquitination after IP Flag in HA-FBXO22 and Flag-VHL overexpression cells. E Western blots detection of HIF-1α-associated ubiquitination after IP Flag in HA-FBXO22 and HIF-1αoverexpression cells.

**Figure S5** HA-Ub-WT, HA-Ub-K6, HA-Ub-K11, HA-Ub-K27, HA-Ub-K29, HA-Ub-K33, HA-Ub-K48, HA-Ub-K63plasmids co-transfected with HA-FBXO22 plasmid and Flag-VHL plasmid into HEK293T cells for 48h, and the cells were then harvested and subjected to Flag IP.

**Figure S6** A-B The migration and invasion abilities of U87-Vector/FBXO22/VHL/FBXO22+VHLwere examined by wound healing and Transwell assay.

**Figure S7** A Western blots was used to detect the protein expression of HIF-1α, FBXO22 in U373-shNC/shFBXO22/ HIF-1α/ shFBXO22+HIF-1α cells. B-G The proliferation, migration and invasion abilities of U373-shNC/shFBXO22/ HIF-1α/ shFBXO22+HIF-1αwere examined by CCK8wound healing Transwell assay and wound healing . All statistical tests were two-sided. ****p*< 0.001.
