# Supplementary figures and images for: FBXO22 promotes glioblastoma malignant progression by mediating VHL ubiquitination and degradation
Source: Cell Death Discov. 2024 Mar 23;10:151. doi: 10.1038/s41420-024-01919-2 (PMC10959977; doi:10.1038/s41420-024-01919-2)

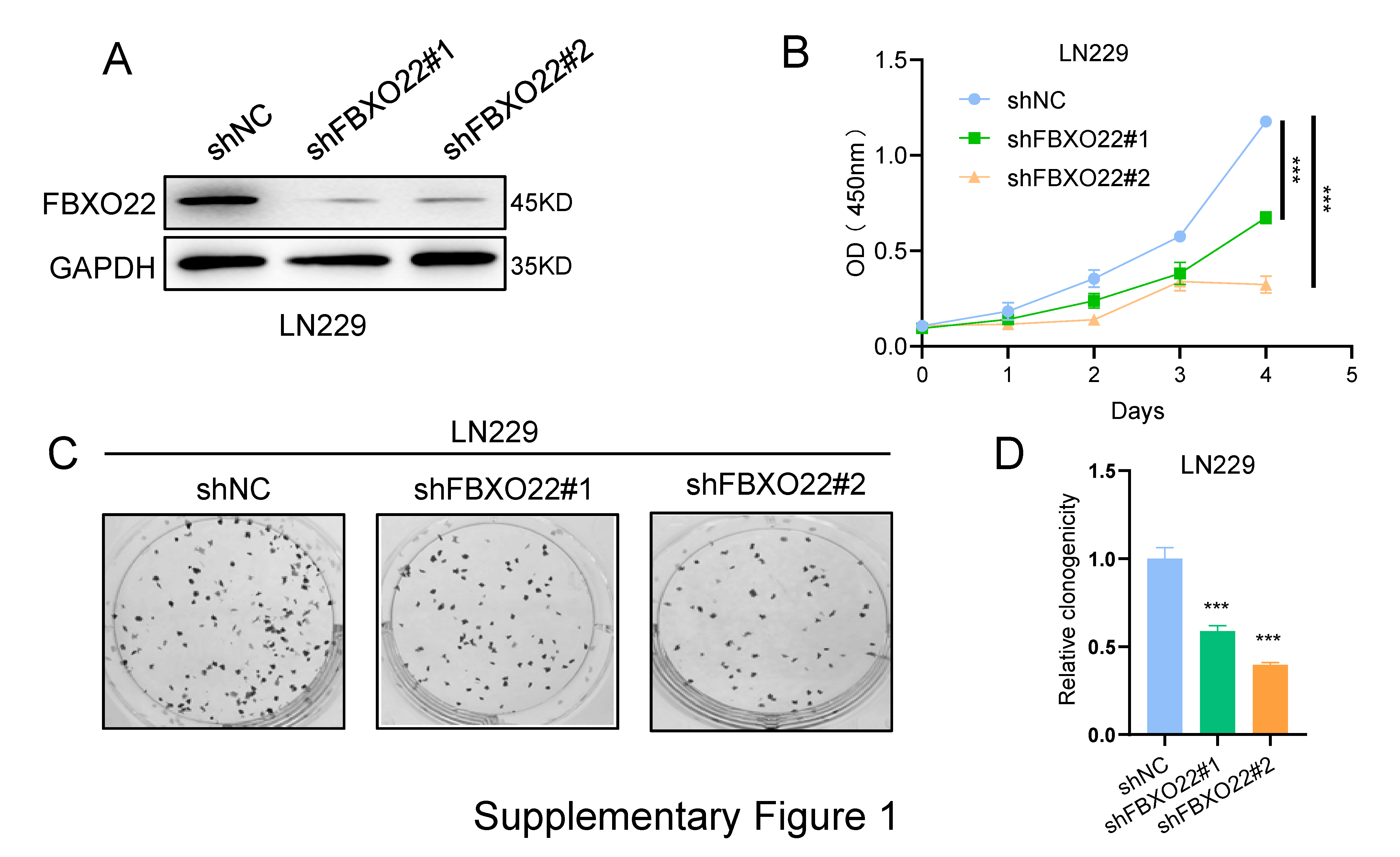

Supplement: Supplementary file 2 — Supplementary Figure 1 [file 41420_2024_1919_MOESM2_ESM.png]

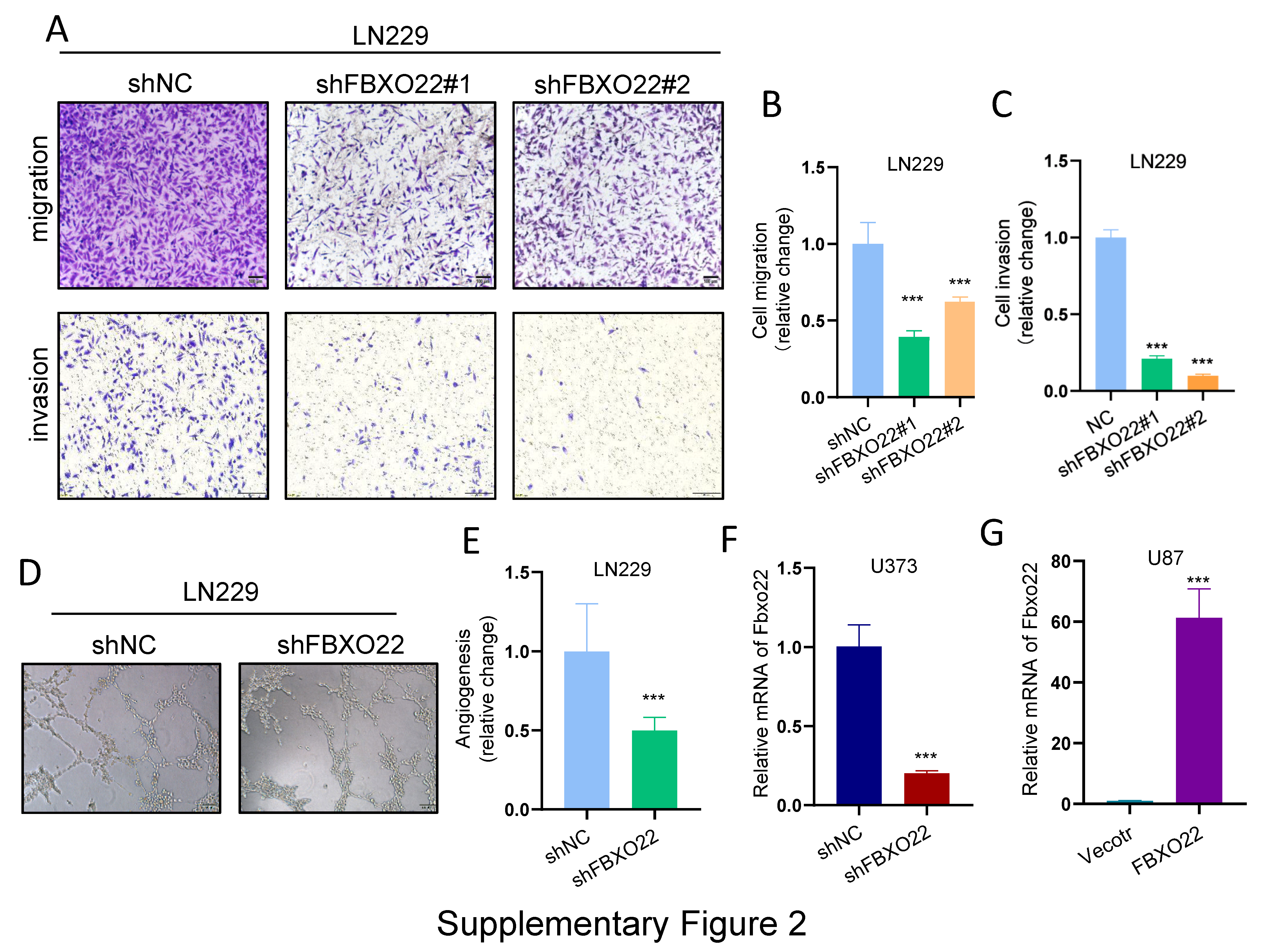

Supplement: Supplementary file 3 — Supplementary Figure 2 [file 41420_2024_1919_MOESM3_ESM.png]

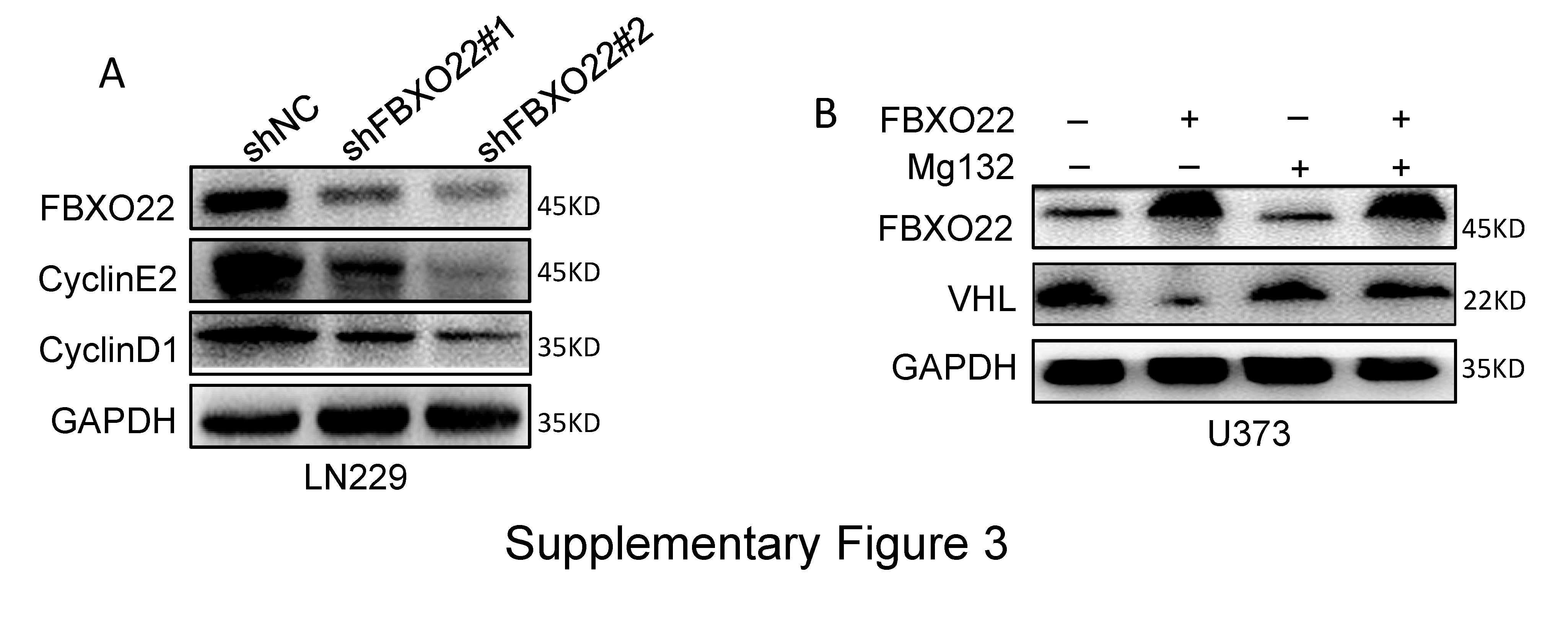

Supplement: Supplementary file 4 — Supplementary Figure 3 [file 41420_2024_1919_MOESM4_ESM.png]

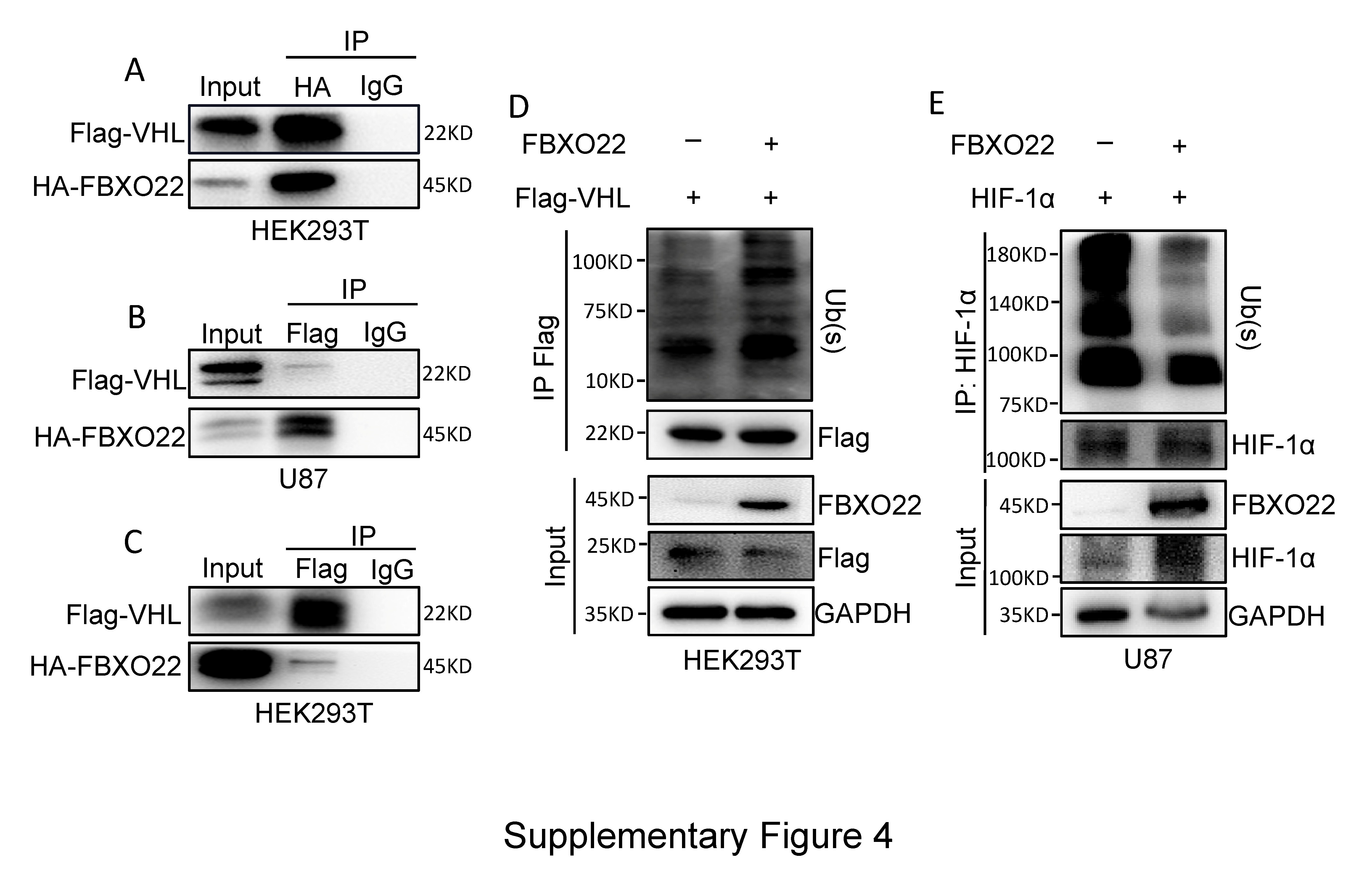

Supplement: Supplementary file 5 — Supplementary Figure 4 [file 41420_2024_1919_MOESM5_ESM.jpg]

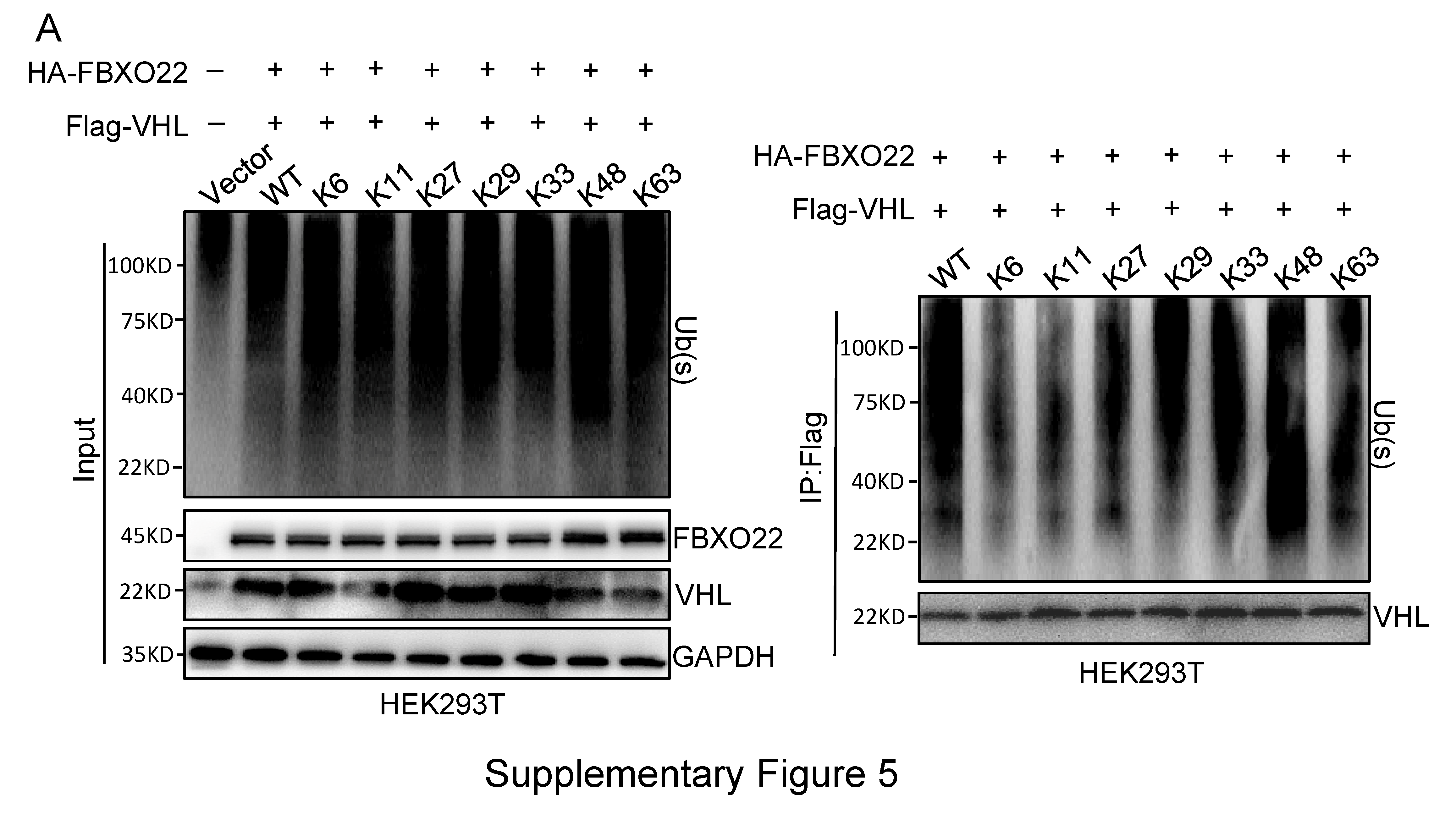

Supplement: Supplementary file 6 — Supplementary Figure 5 [file 41420_2024_1919_MOESM6_ESM.png]

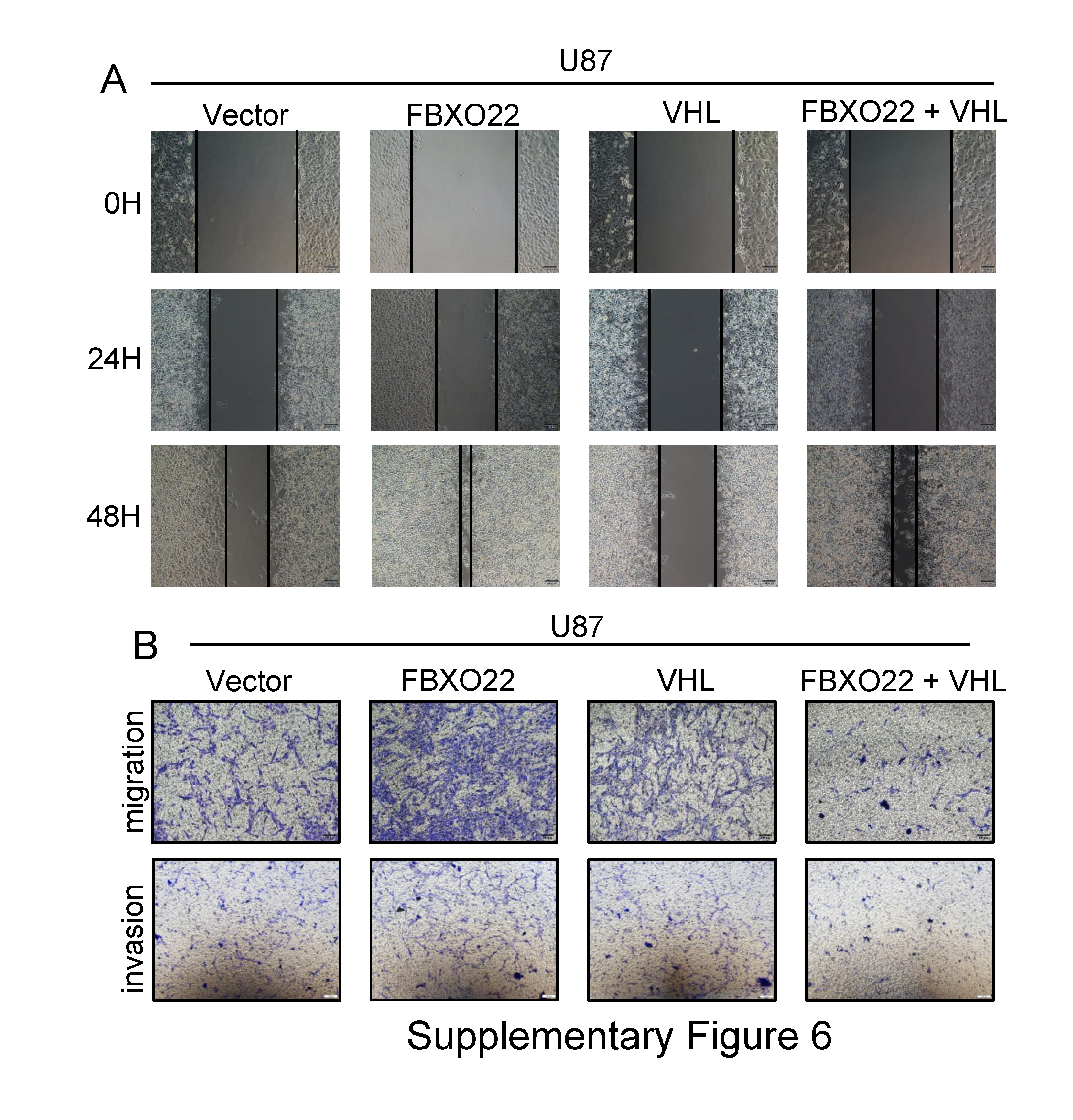

Supplement: Supplementary file 7 — Supplementary Figure 6 [file 41420_2024_1919_MOESM7_ESM.png]

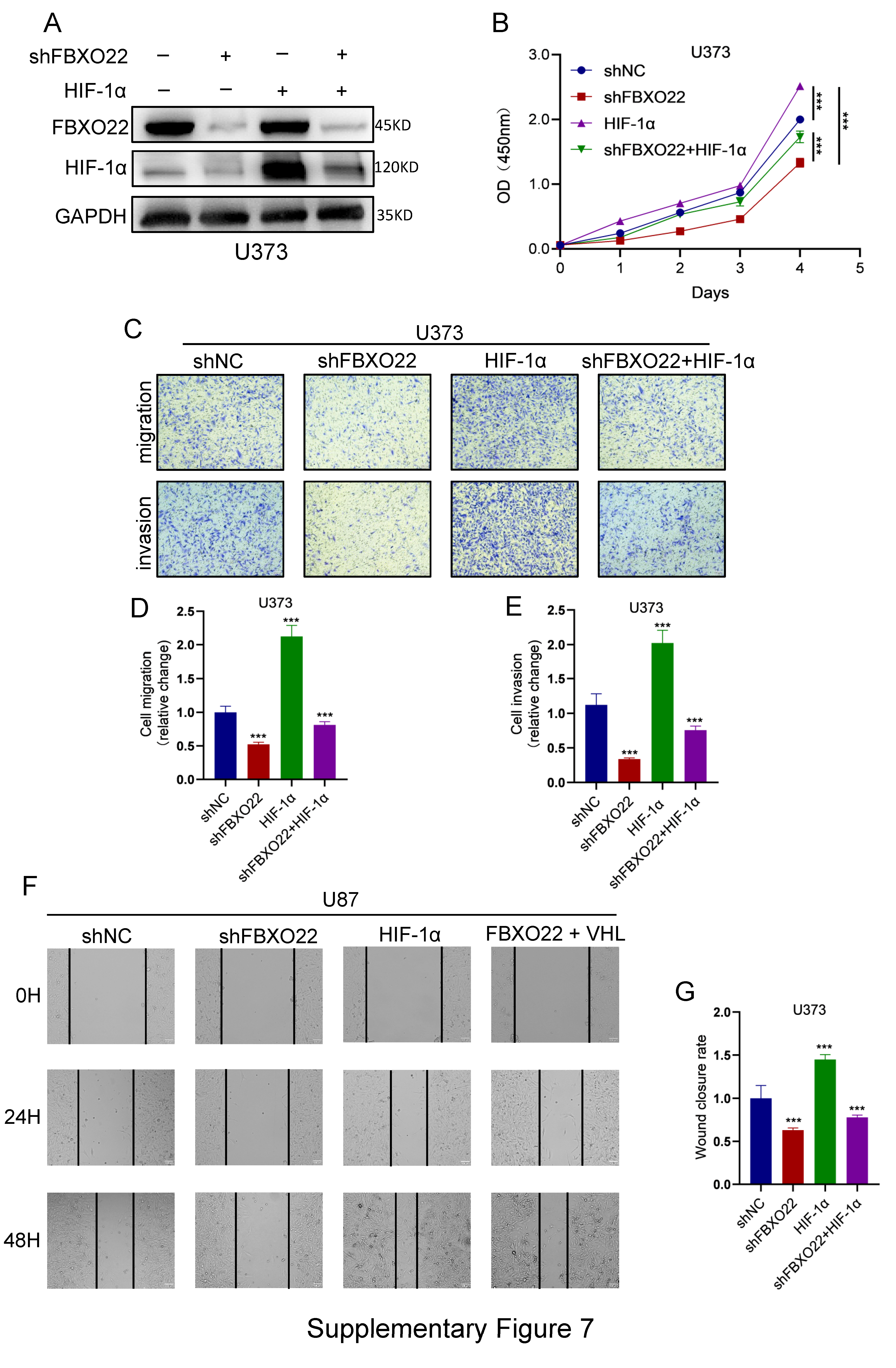

Supplement: Supplementary file 8 — Supplementary Figure 7 [file 41420_2024_1919_MOESM8_ESM.png]
