# Supplementary material for: FBXO22 promotes glioblastoma malignant progression by mediating VHL ubiquitination and degradation
Source: Cell Death Discov. 2024 Mar 23;10:151. doi: 10.1038/s41420-024-01919-2 (PMC10959977; doi:10.1038/s41420-024-01919-2)

Figure 2

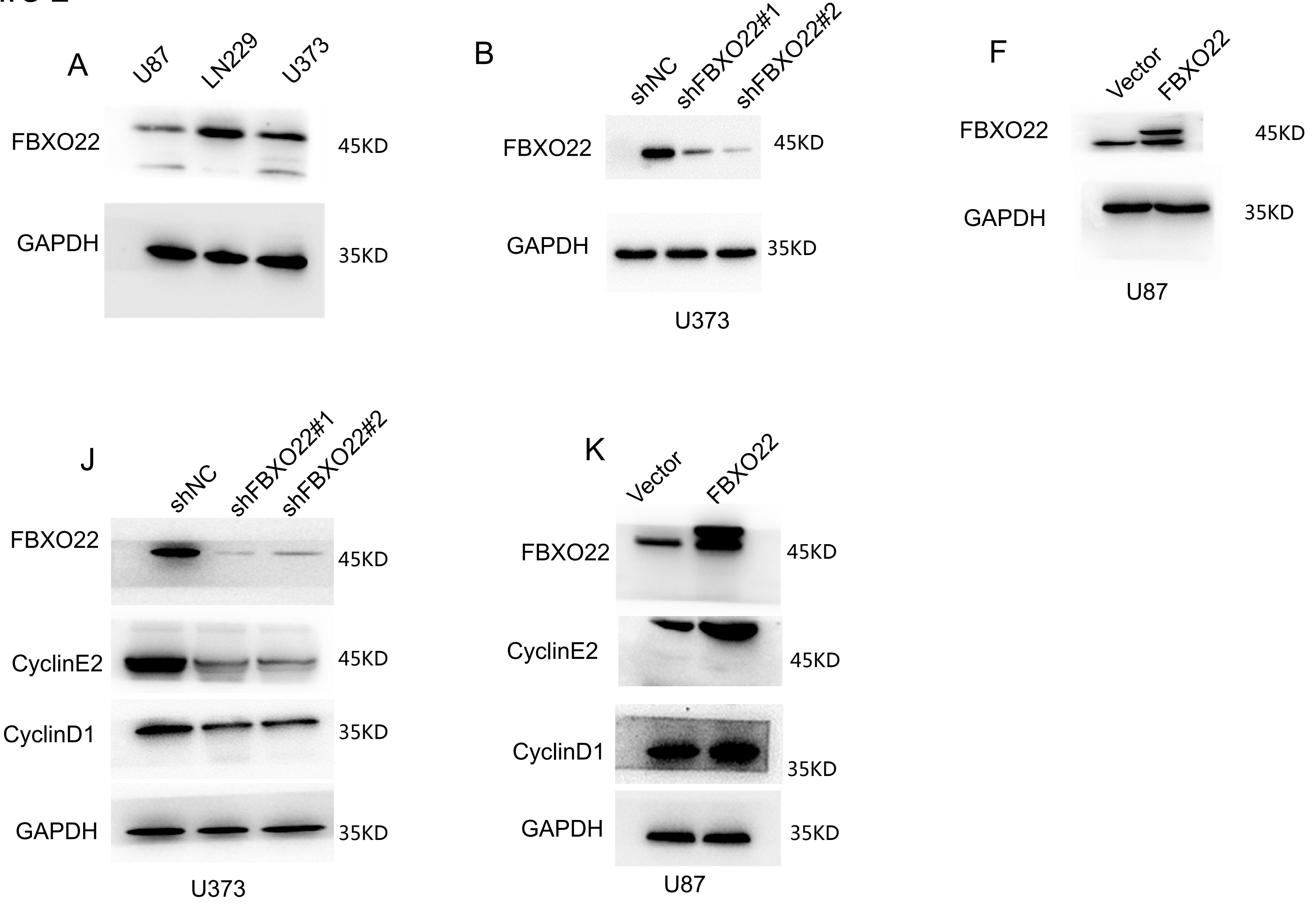
A
U87
LN229
U373
FBXO22
45KD
GAPDH
35KD
B
shNC
shFBXO22#1
shFBXO22#2
FBXO22
45KD
GAPDH
35KD
U373
F
Vector
FBXO22
FBXO22
45KD
GAPDH
35KD
U87
J
shNC
shFBXO22#1
shFBXO22#2
FBXO22
45KD
CyclinE2
45KD
CyclinD1
35KD
GAPDH
35KD
U373
K
Vector
FBXO22
FBXO22
45KD
CyclinE2
45KD
CyclinD1
35KD
GAPDH
35KD
U87

Figure 3

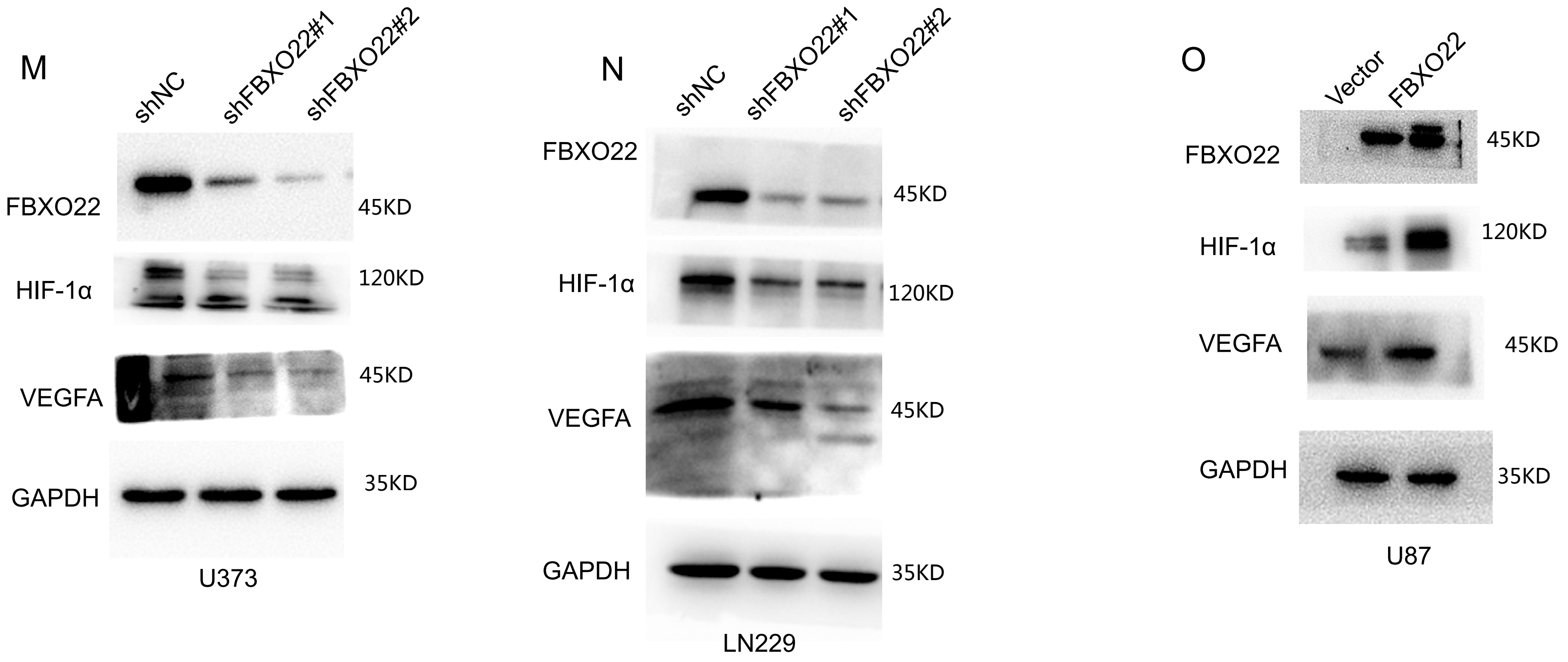
M
shNC
shFBXO22#1
shFBXO22#2
FBXO22
45KD
HIF-1α
120KD
VEGFA
45KD
GAPDH
35KD
U373
N
shNC
shFBXO22#1
shFBXO22#2
FBXO22
45KD
HIF-1α
120KD
VEGFA
45KD
GAPDH
35KD
LN229
O
Vector
FBXO22
FBXO22
45KD
HIF-1α
120KD
VEGFA
45KD
GAPDH
35KD
U87

Figure 4

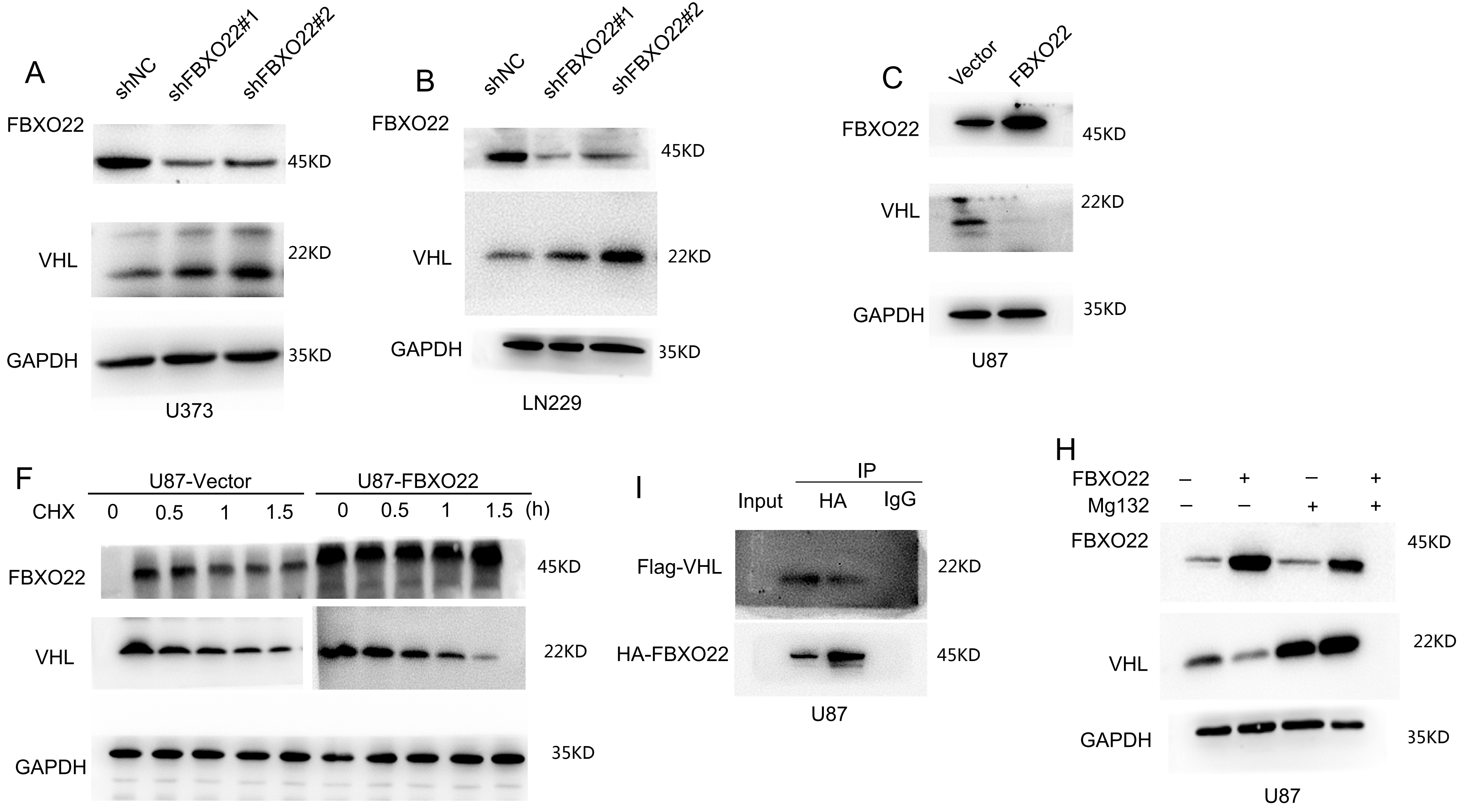
A
shNC
shFBXO22#1
shFBXO22#2
FBXO22
45KD
VHL
22KD
GAPDH
35KD
U373
B
shNC
shFBXO22#1
shFBXO22#2
FBXO22
45KD
VHL
22KD
GAPDH
35KD
LN229
C
Vector
FBXO22
FBXO22
45KD
VHL
22KD
GAPDH
35KD
U87
F
U87-Vector
U87-FBXO22
CHX 0 0.5 1 1.5 0 0.5 1 1.5 (h)
FBXO22
45KD
VHL
22KD
GAPDH
35KD
I
IP
Input HA IgG
Flag-VHL
22KD
HA-FBXO22
45KD
U87
H
FBXO22 – + – +
Mg132 – – + +
FBXO22
45KD
VHL
22KD
GAPDH
35KD
U87

J

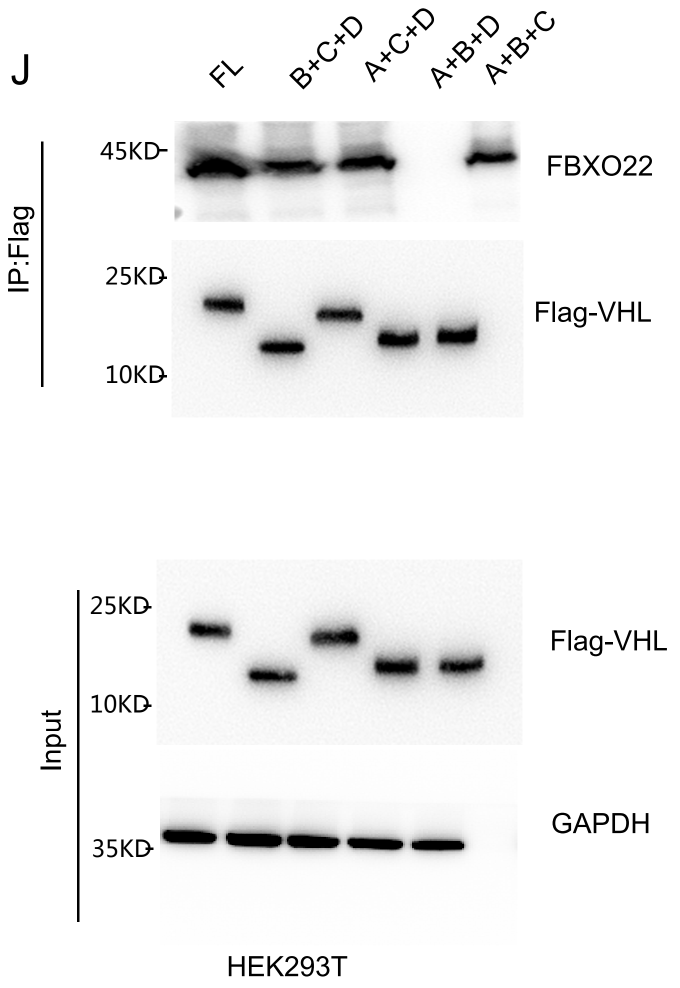

FL
B+C+D
A+C+D
A+B+D
A+B+C
IP:Flag
45KD
FBXO22
25KD
10KD
Flag-VHL
Input
25KD
10KD
Flag-VHL
35KD
GAPDH
HEK293T

L

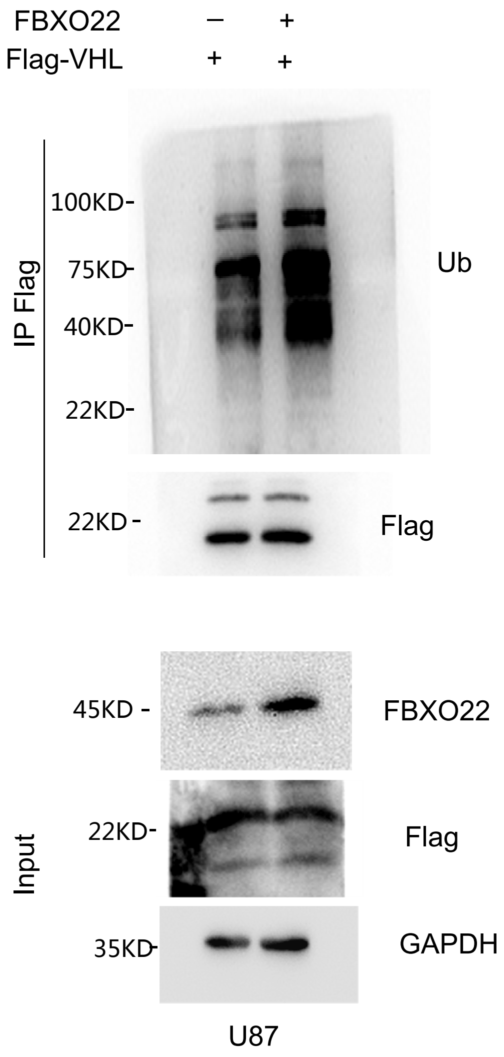

FBXO22 – +
Flag-VHL + +
IP Flag
100KD
75KD
40KD
22KD
Ub
22KD
Flag
Input
45KD
FBXO22
22KD
Flag
35KD
GAPDH
U87

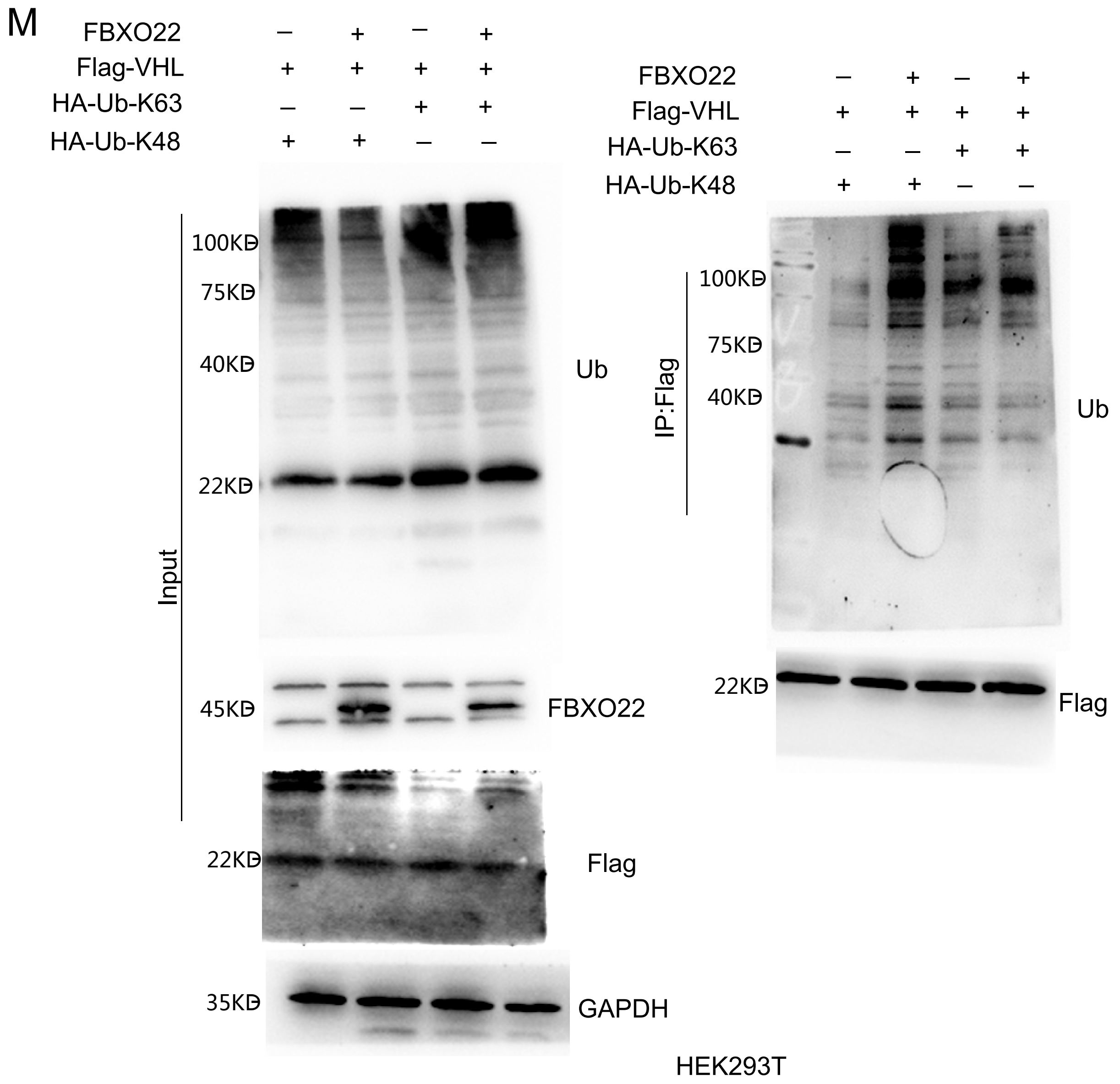
M
FBXO22 – + – +
Flag-VHL + + + +
HA-Ub-K63 – – + +
HA-Ub-K48 + + – –
Input
100KD
75KD
40KD
22KD
Ub
45KD
FBXO22
22KD
Flag
35KD
GAPDH
FBXO22 – + – +
Flag-VHL + + + +
HA-Ub-K63 – – + +
HA-Ub-K48 + + – –
IP:Flag
100KD
75KD
40KD
Ub
22KD
Flag
HEK293T

Figure 5

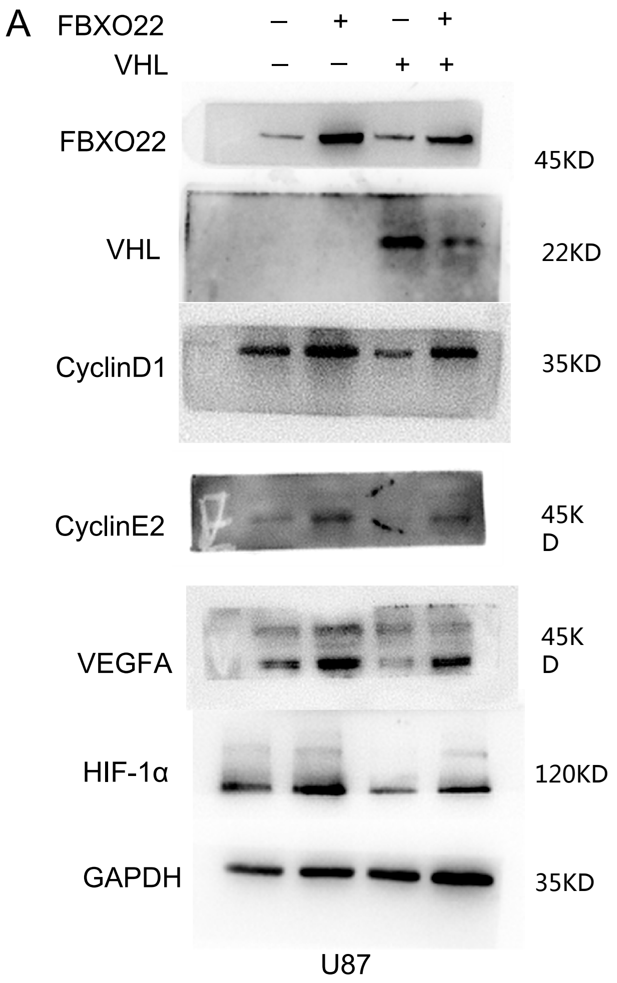
A
FBXO22 – + – +
VHL – – + +
FBXO22
45KD
VHL
22KD
CyclinD1
35KD
CyclinE2
45K
D
VEGFA
45K
D
HIF-1α
120KD
GAPDH
35KD
U87

Supplementary Figure 1

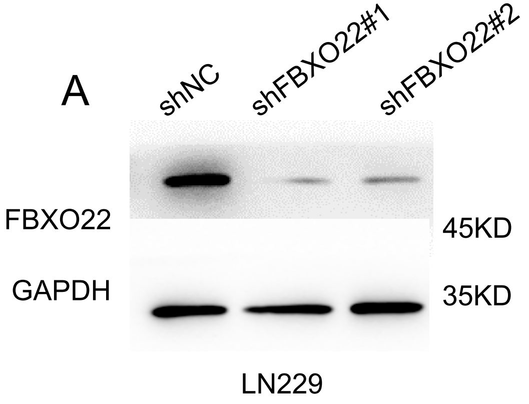

Supplementary Figure 3

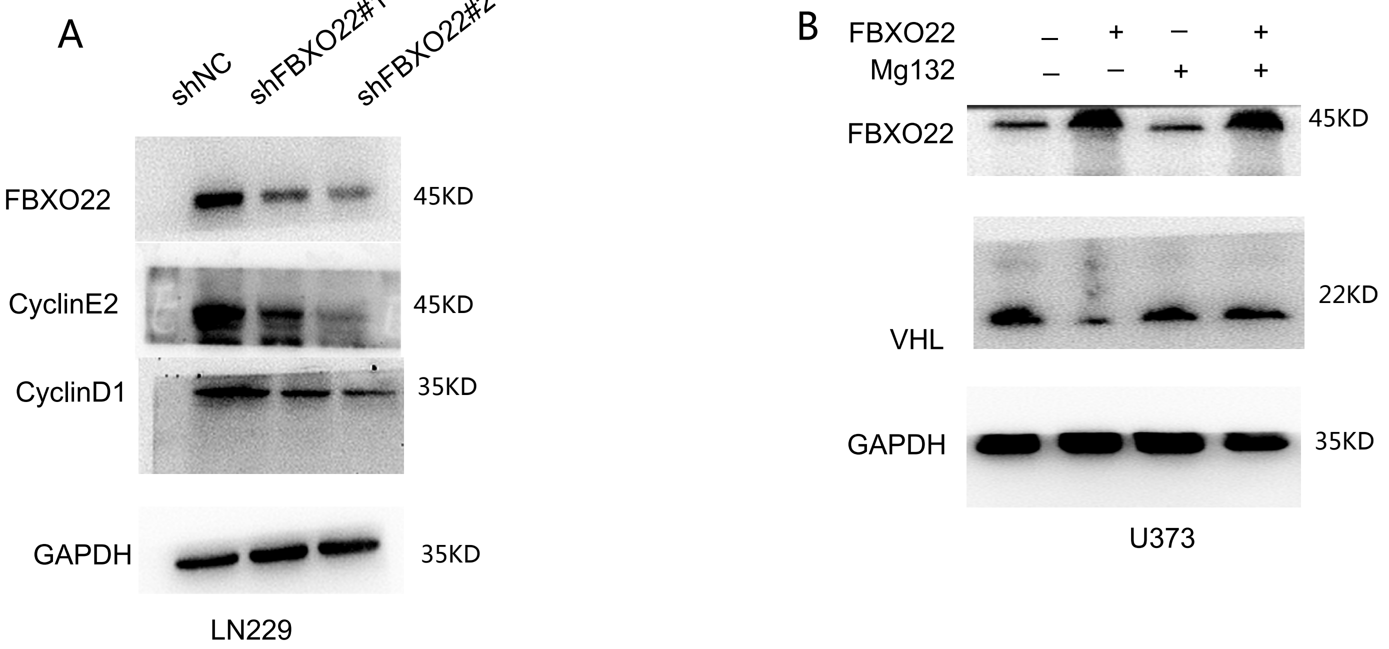

Supplementary Figure 4

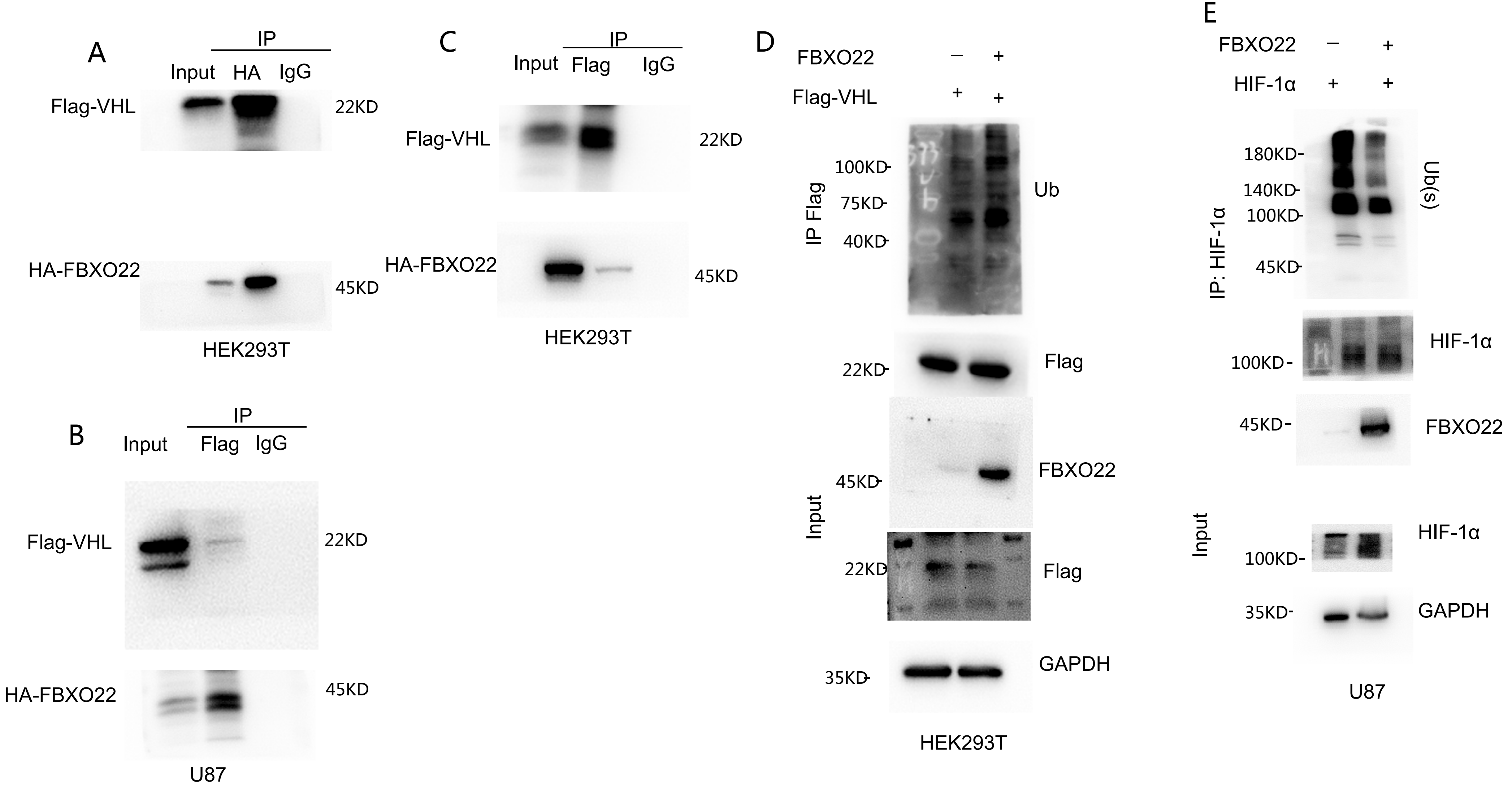

Supplementary Figure 5

A

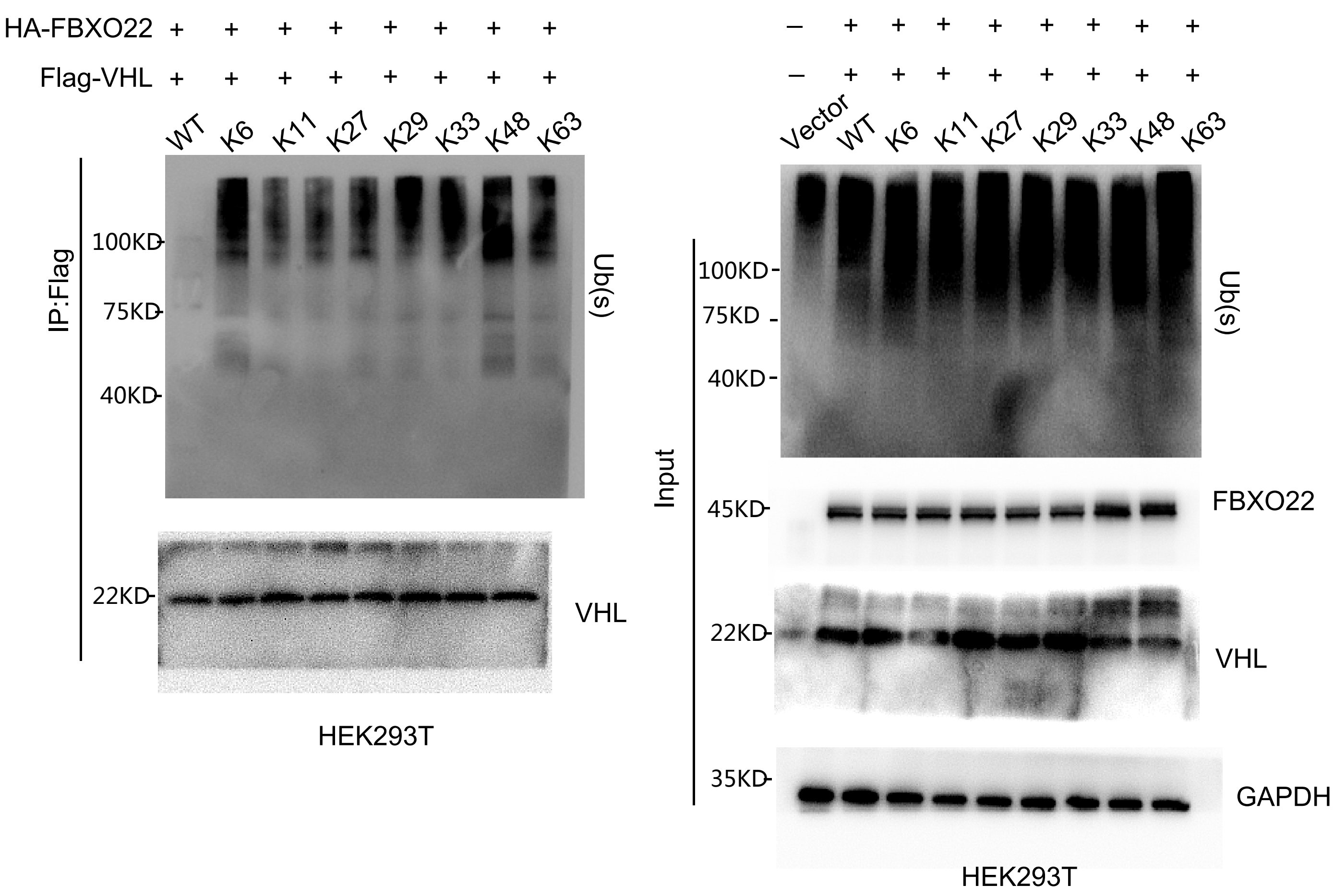

Supplementary Figure 7

A

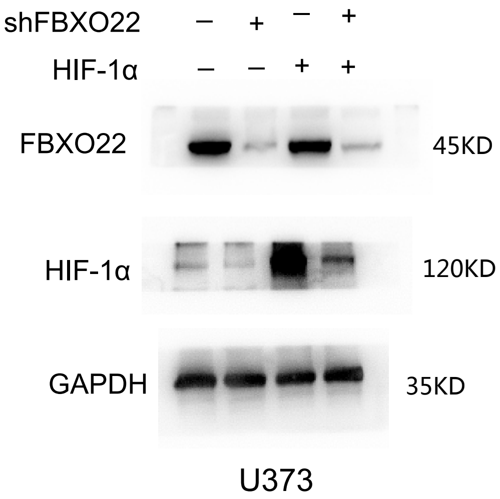

Supplement: Supplementary file 9 — Original Data File [file 41420_2024_1919_MOESM9_ESM.pdf]
